# Investigation of Anticancer Peptides Derived from *Arca* Species Using In Silico Analysis

**DOI:** 10.3390/molecules30071640

**Published:** 2025-04-07

**Authors:** Jixu Wu, Xiuhua Zhang, Yuting Jin, Man Zhang, Rongmin Yu, Liyan Song, Fei Liu, Jianhua Zhu

**Affiliations:** 1 Biotechnological Institute of Chinese Materia Medica, Jinan University, Guangzhou 510632, China; 2 Shandong Engineering Research Center for Efficient Preparation and Application of Sugar and Sugar Complex, Shandong Academy of Pharmaceutical Science, Jinan 250101, China; 3 Shandong Provincial Key Laboratory of Carbohydrate and Glycoconjugate Drugs, Shandong Academy of Pharmaceutical Science, Jinan 250101, China

**Keywords:** proteins from *Arca* species, in silico analysis, bioactive peptides, anticancer

## Abstract

This study employed an integrated in silico approach to identify and characterize anticancer peptides (ACPs) derived from *Arca* species. Using a comprehensive bioinformatics pipeline (BIOPEP, ToxinPred, ProtParam, ChemDraw, SwissTargetPrediction, and I-TASSER), we screened hydrolyzed bioactive peptides from *Arca* species, identifying seventeen novel peptide candidates. Subsequent in vitro validation revealed three peptides (KW, WQIWYK, KGKWQIWYKSL) with significant anticancer activity, demonstrating both high biosafety and clinical potential. Our findings highlight *Arca* species proteins as a valuable source of therapeutic ACPs and establish bioinformatics as an efficient strategy for rapid discovery of bioactive peptides. This approach combines computational prediction with experimental validation, offering a robust framework for developing novel peptide-based therapeutics.

## 1. Introduction

The exponential growth in the global population presents unprecedented challenges to food security, sustainable resource utilization, and healthcare systems worldwide. Marine ecosystems, encompassing approximately 71% of the Earth’s surface, constitute an invaluable repository of biological diversity, including fish, crustaceans, mollusks, and algae species. Throughout human history, the strategic exploitation of these marine resources has played a pivotal role in societal development, offering solutions to nutritional requirements, industrial applications, and therapeutic discoveries. Recent advances in marine biotechnology have further enhanced our capacity to harness these resources, particularly in the development of novel bioactive compounds with pharmaceutical potential [1,2]. Since the discovery of the first marine-derived clinical drug in 1945, marine resources have been utilized in developing anticancer agents, such as Adcetris^®^ and Polivy^®^ [3]. Peptides derived from marine organisms exhibit unique amino acid sequences, which confer distinct biofunctional properties upon enzymatic hydrolysis. This suggests that marine protein hydrolysates may serve as a novel source of pharmacologically active compounds for cancer treatment. Protein hydrolysates, consisting of oligopeptides and free amino acids, demonstrate diverse biological activities, including antioxidant, antiproliferative, antihypertensive, and antimicrobial effects [4,5,6,7]. Enzymatic hydrolysis is hypothesized to release bioactive sites, enhancing the efficacy of polypeptides [8]. Notably, protein hydrolysates from fish, amphibians, and turtles have shown specific antibreast-cancer properties [9]. Marine anticancer peptides (ACPs) modulate multiple cellular and molecular pathways by inducing apoptosis, disrupting cell membranes, altering pH homeostasis, and enhancing immune responses [10]. Thus, marine peptides are valuable tools for discovering novel anticancer therapies and elucidating their mechanisms of action.

The production of bioactive peptides traditionally relies on enzymatic hydrolysis and fermentation, followed by isolation, purification, and screening through techniques such as column chromatography, dialysis, and structural characterization [11]. However, these methods are labor-intensive, time-consuming, and often yield peptides with low purity and efficiency [12]. Advances in bioinformatics have revolutionized this process, enabling rapid and efficient discovery of novel bioactive peptides [13]. Computational tools, including the BIOPEP database and ExPASy peptide cutter, facilitate in silico protein hydrolysis by predicting proteolytic products from known sequences. Additionally, platforms such as PeptideRanker, ToxinPred, Open Targets Platform, SwissTargetPrediction, and I-TASSER are widely used for protein production, activity screening, and applications such as peptide activity prediction, structure–activity relationship analysis, physicochemical property evaluation, and molecular docking [14]. For example, two anticancer oligopeptides (WNHIKRYF, WSVGH) were identified from camel milk protein using quantitative structure–activity relationship (QSAR) modeling and molecular docking [15]. Similarly, 19 ACPs were discovered from the Indian walking catfish (*Clarias magur*) through a three-step computational process involving BIOPEP-UWM for simulated hydrolysis, PeptideRanker for activity scoring, and ToxinPred and AlerPred for property analysis [6]. Additionally, three bioactive peptides (PISLKSE, VSLP, SHTLP) were decoded from walnut protein using SwissADME for target prediction, STRING for protein–protein interaction analysis, and AutoDock Vina 1.1.2 for molecular docking [16].

Marine-derived proteins have gained attention as a promising source for new drug development due to their high biological activity, specificity, and diverse pharmacological properties. *Arca* species, belonging to the *Arca* genus (*Arcidae* family, *Mollusca* phylum, *Lamellibranchiata* class), are marine bivalves that thrive in extreme environments characterized by high salinity, pressure, low temperature, limited light, and nutrient scarcity [17]. These organisms have evolved unique metabolic and defense mechanisms, distinct from terrestrial species, which enable the production of structurally novel and functionally diverse bioactive compounds. As a traditional marine Chinese medicine, *Arca* species are rich in proteins, offering significant potential for developing medicinal functional proteins [18,19]. The medicinal properties of the *Arca* species are primarily attributed to three species: *A. subcrenata*, *A. inflata*, and *A. granosa* [20,21]. Numerous bioactive peptides, including antimicrobial, anticancer, and antioxidant peptides, have been isolated from these species [22]. Several anticancer proteins, such as ASP3, J2-C2, J2-C3, and P6, exhibit potent antitumor activity, with IC50 values ranging from 20 to 1300 µg/mL. For instance, ASP3, derived from A. *subcrenata*, inhibits HepG2 cell proliferation by competitively binding to EGFR [23,24]. J2-C2, isolated from *A. inflata*, induces G2/M phase arrest, significantly suppressing HT-29 cell proliferation [25]. J2-C3, also from *A. inflata*, demonstrates broad antitumor activity, with IC50 values of 65.57, 93.33, and 122.95 µg/mL against A549, HT-29, and HepG2 cells, respectively [26]. P6, another *A. inflata*-derived protein, inhibits colorectal cancer cell proliferation and colony formation by activating the p38-MAPK signaling pathway [27]. These findings highlight the potential of *Arca* species as a valuable source of anticancer peptides. However, research on ACPs derived from these species remains limited, warranting further investigation. Thus, the current study aims to predict and analyze ACPs derived from *Arca*-derived proteins through an in silico approach.

**Figure 1:**
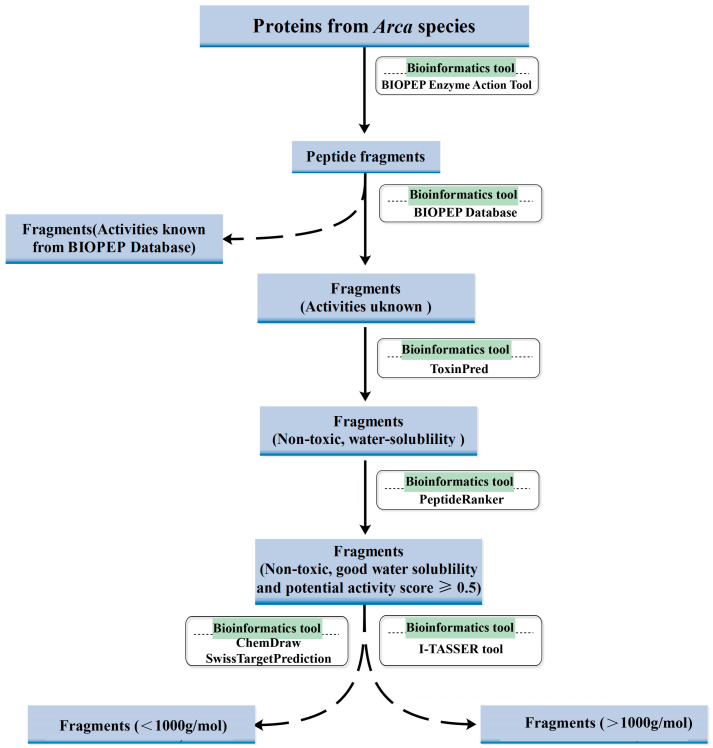
Schematic illustration of the integrated computational pipeline for predicting and screening ACPs derived from *Arca* species. The workflow encompasses (i) in silico enzymatic hydrolysis using BIOPEP-UWM; (ii) bioactivity prediction via PeptideRanker; (iii) physicochemical property evaluation (ToxinPred) and stability assessment (ProtParam); and (iv) structural characterization and functional annotation employing SwissTargetPrediction and I-TASSER tool.

Building upon our previous work identifying anticancer proteins from *A. subcrenata* and *A. inflata* through isolation and characterization, this study pioneers the application of in silico strategies to mine bioactive peptides from *Arca*-derived proteins. We implemented a systematic bioinformatics pipeline (Figure 1), combining BIOPEP-UWM for virtual enzymatic hydrolysis, PeptideRanker for activity scoring, and ToxinPred/ProtParam for safety and physicochemical profiling. Experimental validation revealed three novel peptides—KW, WQIWYK, and KGKWQIWYKSL—exhibiting potent anticancer effects and excellent biosafety. Our results underscore bioinformatics as a high-throughput, resource-efficient platform for bioactive peptide discovery, while solidifying *Arca* proteins as a promising source of ACP candidates. This integrated in silico–in vitro framework not only accelerates therapeutic screening but also opens avenues for sustainable exploitation of marine bioresources in oncological drug development.

## 2. Results and Discussion

### 2.1. In Silico Enzymatic Hydrolysis of ASP3 and J2-C3

Computer-simulated enzymatic hydrolysis is highly effective for modeling interactions between well-characterized proteins and known enzymatic cleavage sites, enabling efficient generation of peptide fragments. Through a comprehensive literature review, we identified four reported *Arca* proteins: ASP3, J2-C2, J2-C3, and P6. P6 was excluded due to its uncharacterized amino acid sequence, while J2-C2 exhibited limited antitumor efficacy (IC50: 1144.26 μg/mL in A549, 1232.28 μg/mL in HepG2, and 1315.71 μg/mL in SPC-A-1), falling below the activity threshold. ASP3 and J2-C3 were selected for further analysis based on their (1) complete sequence availability, (2) <80% sequence homology, and (3) established antitumor activity profiles.

To ensure the analytical relevance, the selected proteins must exhibit distinct structural and functional characteristics. Sequence alignment was used to evaluate the divergence between ASP3 and J2-C3, followed by their amino acid sequence similarity calculation. Homology analysis confirmed that ASP3 and J2-C3 are distinct protein entities.

Given that peptides with specific structural and functional attributes are predominantly encoded within proteins, the BIOPEP-UWM platform was utilized to simulate the enzymatic hydrolysis of ASP3 and J2-C3. Five enzymes—chymotrypsin (EC 3.4.21.1), trypsin (EC 3.4.21.4), pepsin (pH 1.3, EC 3.4.23.1), papain (EC 3.4.22.2), and subtilisin (EC 3.4.21.62)—were selected for in silico enzymatic hydrolysis. Following five iterative cycles of hydrolysis, a total of 205 and 220 peptide fragments were isolated from ASP3 and J2-C3 hydrolysates, respectively.

To identify potentially novel peptides, the derived fragments were cross-referenced with the active peptides cataloged in the BIOPEP database. This comparative analysis involved the exclusion of identical peptide sequences, thereby enabling the identification of unique peptide fragments. Consequently, 100 previously unreported fragments were identified from ASP3, while 103 novel fragments were derived from J2-C3.

**Figure 2:**
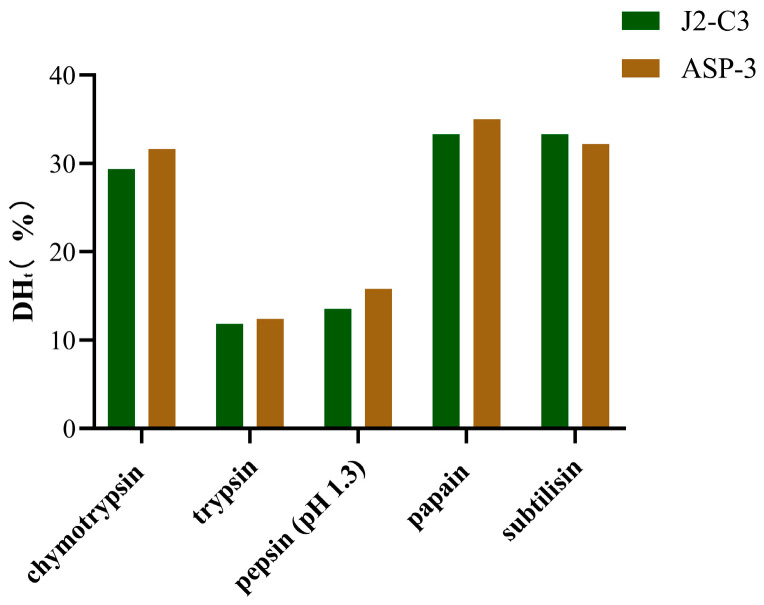
Theoretical degree of hydrolysis by in silico hydrolysis of proteins from *Arca* species using different enzymes.

Furthermore, the theoretical degree of hydrolysis (DHt) was observed to be significantly higher for chymotrypsin (31.6384–29.3785%), papain (35.0282–33.3333%), and subtilisin (33.3333–32.2034%) compared to trypsin (12.4294–11.8644%) and pepsin (pH 1.3) (15.8192–13.5593%) (Figure 2). The elevated DHt values for plant-derived enzymes suggest a higher specificity of these enzymes. Notably, plant proteases exhibited a greater number of cleavage sites (7 or 8) relative to other proteases, which may account for their enhanced hydrolytic efficiency. In contrast, pepsin (pH 1.3) demonstrated specificity for peptide bonds following Phe and Leu residues, while trypsin exclusively targeted bonds subsequent to Lys and Arg residues.

### 2.2. Prediction of Physicochemical Properties of the Peptides

The physicochemical properties and potential toxicity of the aforementioned 203 peptide fragments were subsequently analyzed utilizing the ToxinPred online program. The molecular weight (MW) of the antimicrobial peptides (AMPs) was determined to range from 293.34 to 2404.03 Da. Peptides exhibiting a grand average of hydropathicity (GRAVY) value below 0 and devoid of potential toxicity were selected for further analysis, resulting in the identification of 19 peptide fragments from ASP3 and 70 peptide fragments from J2-C3.

Previous studies have demonstrated that the biological activity of peptide fragments is significantly influenced by their hydrophilic, hydrophobic, and amphiphilic properties [28]. It is well established that the bioavailability of active compounds involves a complex process comprising three distinct phases: absorption, distribution, and metabolism. This necessitates that ACPs must traverse the cellular membrane and reach the intracellular compartment [29]. Considering that the cell membrane consists of a lipid bilayer with hydrophilic exterior surfaces and hydrophobic interior regions, hydrophobicity has been identified as a critical property of ACPs [30]. Additionally, the presence of a biological drug efflux pump, specifically P-glycoprotein (Pgp), has been observed on the cellular membrane [31]. This efflux pump demonstrates a high affinity for hydrophobic (lipophilic) molecules. Consequently, molecules exhibiting excessive hydrophobicity may be subject to efflux by this pump [32]. Therefore, hydrophilicity represents another essential characteristic of ACPs. It is crucial that ACPs maintain optimal amphiphilicity. In peptide analysis, the GRAVY index serves as a valuable parameter for assessing amphiphilicity. This index is calculated as the sum of the hydropathy values of all amino acids in a given sequence divided by the number of amino acids in the sequence [33]. Negative GRAVY values indicate increased hydrophilicity, while positive values denote enhanced hydrophobicity.

### 2.3. Evaluation of Activity Potential of the Peptides

The aforementioned peptides were subjected to scoring utilizing the PeptideRanker computational tool. A total of ten and fourteen peptides exhibiting potential bioactivity (with a score ≥ 0.5) were identified from the ASP3 and J2-C3 datasets, respectively.

The prediction and subsequent verification of bioactivity for individual peptide fragments represented a substantial research endeavor, characterized by significant resource allocation and suboptimal efficiency. In the context of rapid advancements in bioinformatics methodologies, PeptideRanker has emerged as a sophisticated neural network-based predictive platform for biopeptide activity assessment, offering substantial temporal efficiency [34]. This computational tool was developed through the integration of experimental data derived from empirically validated bioactive peptides. When processing user-submitted peptide sequences, PeptideRanker employs an algorithmic transformation of these sequences into feature vectors, subsequently scoring them through a pre-trained predictive model. The resultant scores demonstrate a positive correlation with predicted bioactivity levels, thereby enabling researchers to prioritize peptide sequences for subsequent empirical validation based on their relative ranking [35,36]. However, it is crucial to note that this predictive framework is limited to the assessment of activity potential and does not provide quantitative evaluation of activity magnitude or potency.

### 2.4. Stability Assessment of Peptides from Arca Species

To assess the stability of peptide fragments, the ProtParam tool was utilized to evaluate specific physicochemical parameters, with the aim of identifying and eliminating unstable peptides. This process resulted in the retention of 7 and 13 peptide fragments from ASP3 and J2-C3, respectively, for subsequent predictive analyses. Upon integration of the datasets, a total of 17 peptides exhibiting potential biological activity were identified (Table 1).

Stability has been established as a critical determinant for the efficacy of ACPs. Computational predictions indicate that peptide instability may lead to diminished bioavailability and reduced half-life. The ProtParam tool, which was specifically developed to compute a comprehensive array of physical and chemical properties for protein sequences [37,38], accepts input in the form of UniProtKB accession numbers (AC), identifiers (ID), or raw amino acid sequences (with spaces and numerical characters automatically excluded). The calculated parameters encompass molecular weight, theoretical isoelectric point (pI), amino acid composition, atomic composition, estimated half-life, instability index, aliphatic index, and GRAVY. For instance, the ProtParam online tool has been effectively employed to determine various physicochemical properties of FAD3 sequences in linseed protein, including molecular weight, pI, estimated half-life, instability index, and other relevant characteristics [39].

### 2.5. Activity Prediction of Peptides from Arca Species

#### 2.5.1. Activity Prediction of the Peptides (<1000 g/mol)

**Figure 3:**
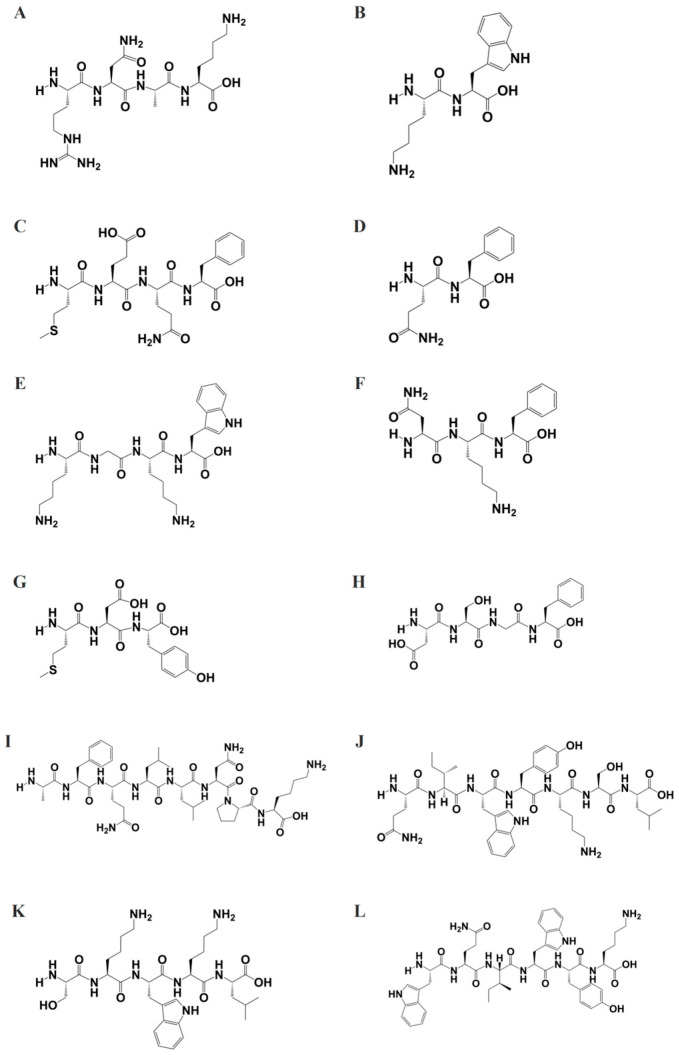
Predicted structures of peptides (molecular weight < 1000 g/mol) generated using ChemDraw software. Shown are the structures of RNKF (**A**), KW (**B**), MEQF (**C**), QF (**D**), KGKW (**E**), NKF (**F**), MDY (**G**), DSGF (**H**), AFQLLNPL (**I**), QIWYKSL (**J**), SKWKL (**K**), and WQIWYK (**L**).

**Figure 4:**
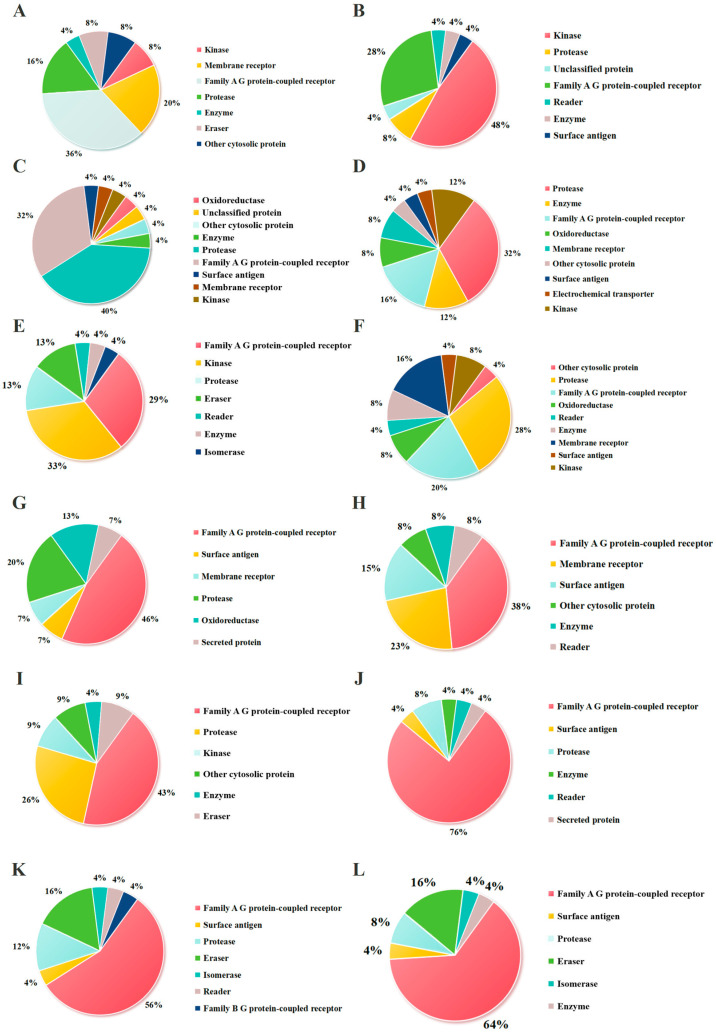
Classes of proteins (according to the classification provided by SwissTargetPrediction) potentially interacting with RNKF (**A**), KW (**B**), MEQF (**C**), QF (**D**), KGKW (**E**), NKF (**F**), MDY (**G**), DSGF (**H**), AFQLLNPL (**I**), QIWYKSL (**J**), SKWKL (**K**), and WQIWYK (**L**). The detailed information is shown in Supplementary Tables S1–S12.

To further elucidate the biological activity of peptide fragments with molecular weights below 1000 g/mol, the structures of these peptides were generated using ChemDraw software (Figure 3) and saved in SMILES format. Subsequently, these structures were subjected to target prediction analysis utilizing the SwissTargetPrediction tool. The SwissTargetPrediction analysis generated two complementary datasets: (1) a hierarchical target classification profile (Figure 4), illustrating the distribution of predicted targets across major biological classes, and (2) detailed target-specific information (Supplementary Tables S1–S12), including probability scores for each candidate target.

As shown in Figure 4, the potential targets of the peptide fragments primarily fall into three categories: kinases, membrane receptors, and Family A G protein-coupled receptors (GPCRs). It has been reported that kinases have been widely recognized in the development of antitumor drugs. Kinases play a vital role in the apoptosis, proliferation, differentiation, migration, and cell cycle of tumor cells [40], such as cyclin-dependent kinases (CDK), ephrin type-A receptors (EPHA), and ephrin type-B receptors (EPHB). The results showed that the proportions of kinases in the potential targets were RNKF (8%), KW (48%), MEQF (4%), QF (12%), KGKW (33%), NKF (8%), MDY (0), DSGF (0), AFQLLNPL (9%), QIWYKSL (0), SKWKL (0), and WQIWYK (0). Membrane receptors, as critical functional components of the cell membrane, play essential roles in fundamental biological processes including cellular signal transduction, molecular transport, and intercellular interactions [41]. In the context of oncology, the association between membrane receptors and tumorigenesis is particularly significant, as their aberrant expression or functional impairment directly or indirectly contributes to tumor initiation and progression [42]. Transforming growth-factor beta (TGF-β), integrin beta (ITGB), and integrin alpha (ITGA) are currently being extensively investigated membrane protein targets in antitumor therapeutic research. As demonstrated in Figure 4, the proportions of membrane receptors among potential targets were RNKF (20%), KW (0), MEQF (4%), QF (8%), KGKW (0), NKF (16%), MDY (7%), DSGF (15%), AFQLLNPL (0), QIWYKSL (0), SKWKL (0), and WQIWYK (0). Moreover, G protein-coupled receptors (GPCRs) represent a class of transmembrane signaling proteins that orchestrate extracellular signal transduction at the plasma membrane [43]. GPCRs interact with diverse agonist ligands encompassing bioactive small molecules, lipid mediators, peptide hormones, and proteinaceous ligands, thereby regulating fundamental cellular processes including metabolic regulation, homeostatic maintenance, and chemotactic responses [44]. Dysregulation of GPCR signaling is associated with numerous clinical conditions, especially cancer. For example, the functional significance of GPCRs in metastatic progression is exemplified by the upregulation of C-X-C chemokine receptor type 4 (CXCR4) and C-C chemokine receptor type 7 (CCR7) in breast cancer, where their overexpression facilitates organ-specific metastasis to lung and bone marrow tissues that exhibit elevated expression of corresponding chemokine ligands [45]. The results showed that the proportions of G protein-coupled receptors among potential targets were RNKF (36%), KW (28%), MEQF (4%), QF (16%), KGKW (29%), NKF (20%), MDY (46%), DSGF (38%), AFQLLNPL (43%), QIWYKSL (76%), SKWKL (56%), and WQIWYK (64%). The proportion of these three categories of targets is of great significance for judging whether the peptide has antitumor activity. The cumulative proportions of these three target categories (a key indicator of potential antitumor activity) were RNKF (64%), KW (76%), MEQF (12%), QF (36%), KGKW (62%), NKF (44%), MDY (53%), DSGF (53%), AFQLLNPL (52%), QIWYKSL (76%), SKWKL (56%), and WQIWYK (64%).

To efficiently evaluate peptide activity across diverse protein categories, we conducted an in-depth analysis of specific targets within the three major classes. Our findings demonstrate that RNKF shows particularly promising inhibitory activity against three key therapeutic targets: CDK, TGB, and CXCR4 (Supplementary Table S1). According to the Open Targets Platform, RNKF may hold therapeutic promise for breast cancer, chronic lymphocytic leukemia, small-cell lung cancer, acute myeloid leukemia, glioblastoma multiforme, central nervous system cancers, and melanoma. Furthermore, the potential targets of KW were identified as ephrin type-A receptors 4-8 (EPHA4-8), ephrin type-B receptors 2-6 (EPHB2-6), and somatostatin receptors 1-5 (SSTR1-5) (Supplementary Table S2). These findings suggest that KW may be a viable therapeutic candidate for chronic myelogenous leukemia, acute lymphoblastic leukemia, colorectal cancer, and neuroendocrine tumors.

The structural configuration of MEQF was found to be analogous to that of QF, with both peptides sharing similar targets, including ITGB, ITGA, and CDK (Supplementary Tables S3 and S4). This similarity suggests that MEQF may be effective in treating conditions such as glioblastoma multiforme, central nervous system cancers, melanoma, and breast cancer. KGKW, which shares structural similarities with KW, was predicted to target EPHA, EPHB, and histone deacetylases (HDAC) (Supplementary Table S5). These targets indicate potential efficacy in treating multiple myeloma, primary myelofibrosis, T-cell non-Hodgkin lymphoma, chronic myelogenous leukemia, acute myeloid leukemia, lymphocytic leukemia, and colorectal cancer. Similarly, DSGF, RNKF, and NKF were observed to target the same proteins (ITGB and ITGA) due to their structural similarities (Supplementary Tables S6 and S7), suggesting potential applications in glioblastoma multiforme, central nervous system cancers, and melanoma. Additionally, the majority of MDY’s potential targets were associated with analgesia and sedation (Supplementary Table S8).

Peptides AFQLLNPK, QIWYKSL, SKWKL, and WQIWYK were predicted to target EPHA, EPHB, and HDAC, indicating potential therapeutic applications in multiple myeloma, primary myelofibrosis, T-cell non-Hodgkin lymphoma, chronic myelogenous leukemia, acute myeloid leukemia, lymphocytic leukemia, and colorectal cancer (Supplementary Tables S9–S12).

These results collectively demonstrated that these peptide fragments might have potential antitumor activity. The SwissTargetPrediction tool, designed to predict the most likely protein targets of small molecules, is particularly suitable for compounds with molecular weights below 1000 g/mol [46,47]. The tool operates on the principle of similarity-based reverse screening, comparing input molecules against a database of 376,342 compounds experimentally active against 3068 macromolecular targets [48]. By facilitating the identification of potential targets, the SwissTargetPrediction tool significantly reduces the experimental workload and enables researchers to focus their investigations more efficiently.

#### 2.5.2. Activity Structure Prediction of the Peptides (>1000 g/mol)

To investigate the bioactivity of peptide fragments with molecular weights exceeding 1000 g/mol, the three-dimensional structures, potential molecular targets, and functional properties of these peptides were computationally predicted using the I-TASSER tool. The I-TASSER tool, a sophisticated online platform, leverages cutting-edge algorithms to predict protein structures and functions [49]. This platform not only automates the generation of high-quality three-dimensional models from amino acid sequences but also aids in the prediction of biological functions [50,51]. This tool is particularly suitable for analyzing peptide fragments comprising more than 10 amino acids.

**Figure 5:**
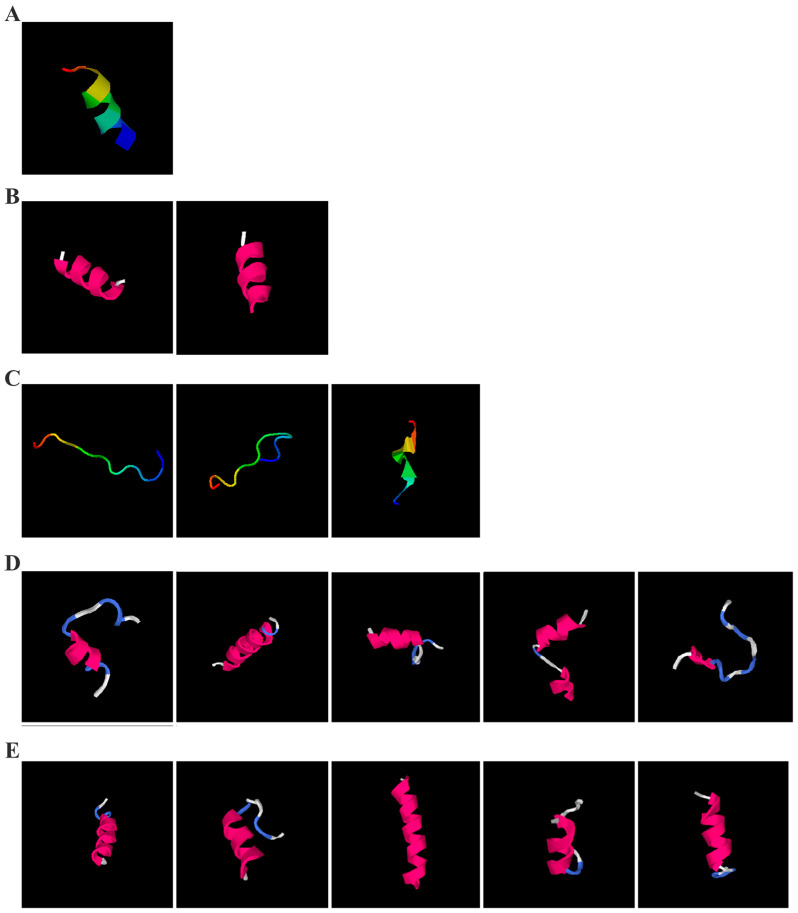
Three-dimensional structural prediction of peptides (molecular weight > 1000 g/mol) by the the I-TASSER algorithm: DMESWWKKYVF (**A**), VDMESWWNTYIFK (**B**), KGKWQIWYKSL (**C**), GDKASGVKVDMESWWNTYIF (**D**), and GDKAQGVKTDMESWWKKYVF (**E**).

As shown in Figure 5A, the predicted three-dimensional structure of DMESWWKKYVF exhibits a single conformation characterized by the presence of an α-helix. Subsequent analysis using the I-TASSER tool, which performed structural alignment and confidence scoring, identified three potential binding targets: Propionyl-CoA carboxylase, Acetyl-CoA carboxylase 2, and the Plasmodium falciparum Atg8-Atg3 peptide complex. These findings suggested that DMESWWKKYVF might have the ability to interfere with cellular metabolic processes and resist malaria (Table 2). Furthermore, predictive modeling using the I-TASSER tool generated two plausible three-dimensional conformations for the peptide VDMESWWNTYIFK (Figure 5B). Functional annotation revealed four potential molecular targets: RNA-binding protein, transferase/lipid-binding protein, photosynthetic enzymes, and the transport protease complex of Plasmodium falciparum Atg8-Atg3. These predictions suggested that VDMESWWNTYIFK might possess multiple biological functions, including modulation of protein synthesis, antimalarial activity, and regulation of lipid metabolism (Table 3).

Structural analysis revealed that the peptide KGKWQIWYKSL predominantly adopts a random coil conformation in its secondary structure (Figure 5C). Functional prediction through I-TASSER-based screening identified three potential molecular targets: the nucleoprotein of Mycobacterium tuberculosis H37Rv, lectin, and hydrolase from Dictyostelium discoideum. These findings suggested that KGKWQIWYKSL might function as an antibacterial agent or immune modulator (Table 4). Moreover, structural prediction by the I-TASSER tool identified five distinct three-dimensional conformations of the peptide GDKASGVKVDMESWWNTYIF (Figure 5D). Functional annotation through structural homology analysis against the PDB database revealed five potential molecular targets: DNA transcriptase, α-galactosidase, helicobacter pylori type II dehydroquinase, human neuronal calcium sensor 1 (NCS1), and procaspase-6. These findings suggested that GDKASGVKVDMESWWNTYIF might possess multiple biological functions, including induction of apoptosis, modulation of protein expression, anti-Helicobacter pylori activity, and regulation of glucose metabolism (Table 5). Also, structural analysis of GDKAQGVKTDMESWWKKYVF using the I-TASSER tool (Figure 5E) generated five predicted conformations. Functional annotation identified five potential molecular targets: HIV-1 integrase, photosynthetic enzymes, carboxyvinyl-carboxyphosphonate phosphorylmutase, and the 70S ribosome. These predictions suggested that GDKAQGVKTDMESWWKKYVF might exhibit multiple biological activities, including interference with protein synthesis, anti-HIV properties, antibacterial effects, and antioxidant capacity (Table 6).

The results demonstrated that KGKWQIWYKSL, GDKASGVKVDMESWWNTYIF, VDMESWWNTYIFK, GDKAQGVKTDMESWWKKYVF, and DMESWWKKYVF exhibited distinct secondary structural characteristics, particularly α-helical conformations. Moreover, these peptide fragments shared the potential ability to influence cellular energy metabolism. Cancer cells predominantly rely on glycolysis for energy production, necessitating increased glucose uptake, which represents a potential target for antitumor strategies. Certain pharmacological agents, such as 2-chlorobenzylphenol, PDH inhibitors, metformin, and *Aspergillus*-derived compounds, may disrupt glycolysis or promote fatty acid oxidation, thereby depriving tumor cells of energy and inducing cell death [52]. Lipid metabolism also plays a critical role in tumor cell survival. Antitumor agents like rosuvastatin may exert their effects by modulating lipid metabolism, such as promoting fatty acid degradation or inhibiting lipid storage [53]. Nucleic acid metabolism further contributes to energy regulation, as protein synthesis is an energy-intensive process. Antitumor drugs such as carfilzomib, ixazomib, and ammonium formate may impair ribosome function, reducing protein synthesis and inhibiting tumor cell proliferation [54]. Additionally, the metabolic dependencies of tumor cells may present specific therapeutic targets. For instance, inhibition of key enzymes or metabolic pathways may effectively disrupt energy supply and impair tumor growth [55].

ACPs are a class of amino acid-based molecules with specific structural features that enable binding to tumor cell surface antigens and exerting biological effects. The secondary structure of peptides, including α-helices and β-sheets, is critical for their functional properties. The α-helix, typically located in hydrophobic regions, contributes to peptide stability, while β-sheets confer structural rigidity and influence solubility and binding affinity [56,57]. The spatial orientation of peptides may enhance their surface activity, facilitating targeted interactions with cancer cell membranes. The angle of interaction can destabilize lipid packing in the membrane, promoting membrane penetration.

Post-translational modifications (PTMs), including glycosylation, tyrosine phosphorylation, and palmitoylation, can significantly alter protein function and cellular signaling pathways. These chemical modifications may exert antitumor effects through multiple mechanisms. Glycosylation modulates cell surface receptor activity and immune recognition [58]. Tyrosine phosphorylation controls critical pathways in cell cycle regulation and apoptosis [59]. Palmitoylation regulates membrane localization and signaling protein trafficking [60]. These modifications represent potential strategies for enhancing the efficacy of natural peptides. The findings underscore the importance of secondary structure in mediating the interaction between ACPs and cancer cell membranes, ultimately inducing membrane disruption and cell death [61].

By employing these computational and structural analyses, the study provides insights into the potential mechanisms of action and therapeutic applications of the investigated peptide fragments, highlighting their relevance in targeting cancer cell metabolism and membrane interactions.

### 2.6. Evaluation of Anticancer Activity

For efficient bioactivity screening, we selected the six highest-scoring peptides based on their PeptideRanker scores and prepared them as 100 mg/mL standardized aqueous solutions for experimental validation.

**Figure 6:**
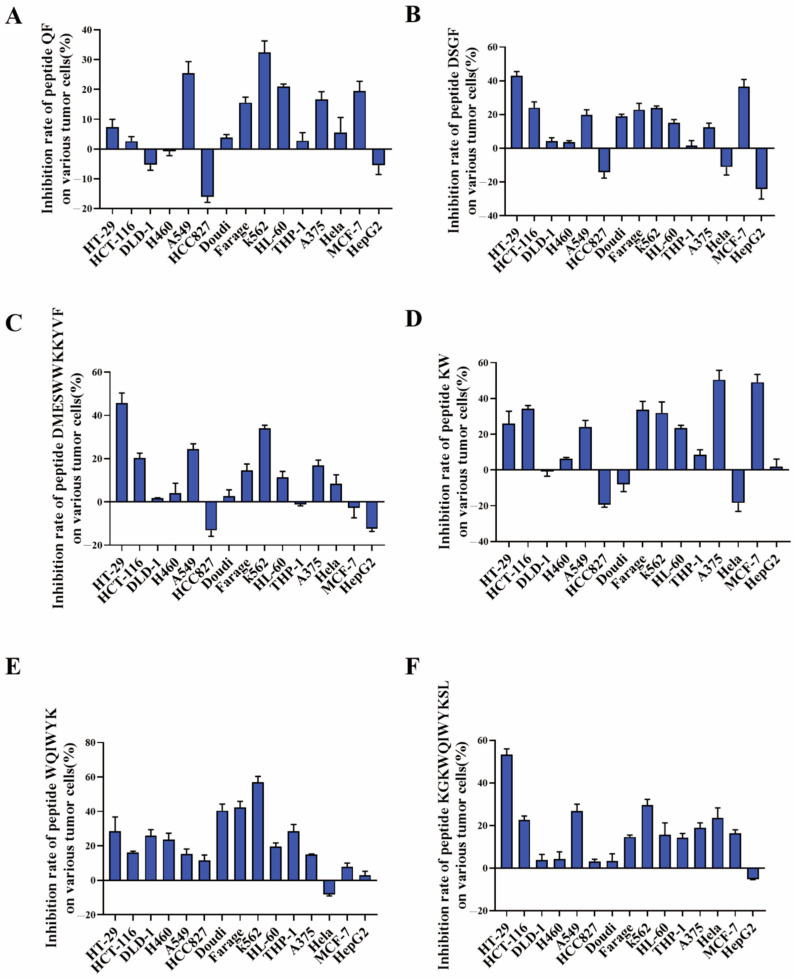
Cytotoxicity evaluation of peptide fragments QF (**A**), DSGF (**B**), DMESWWKKYVF (**C**), KW (**D**), WQIWYK (**E**), and KGKWQIWYKSL (**F**) against multiple tumor cell lines. Results are expressed as mean ± standard deviation from three independent experiments (n = 3).

As demonstrated in Figure 6, the inhibitory effects of peptides QF, DSGF, and DMESWWKKYVF on tumor cells did not meet anticipated outcomes, with inhibition rates below 50% (Figure 6A–C). Although DMESWWKKYVF exhibited near 50% inhibition against HT-29 cells, its poor aqueous solubility posed formulation challenges for further development. These results indicated that peptide fragments identified through in silico analysis necessitate further validation. In contrast, peptide KW exhibited a inhibition rate of 50.44% against A375 cells (Figure 6D), while peptide WQIWYK demonstrated a 56.9% inhibition rate against K563 cells (Figure 6E). Additionally, peptide KGKWQIWYKSL showed a 53.33% inhibition rate against HT-29 cells (Figure 6F). Collectively, KW, WQIWYK, and KGKWQIWYKSL showed relatively stronger anticancer activity, suggesting they might represent more promising leads for subsequent optimization studies compared to other candidates. Specifically, KW effectively inhibited A375 cell growth, WQIWYK suppressed K563 cell proliferation, and KGKWQIWYKSL inhibited HT-29 cell growth. The combined use of computational simulation and experimental validation significantly improves screening efficiency over traditional methods, enabling faster discovery of multiple antitumor peptides.

**Figure 7:**
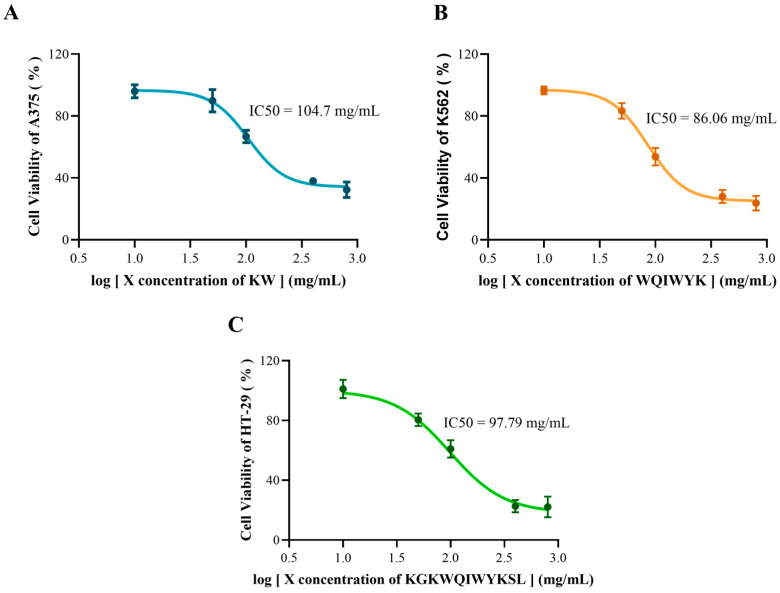
Cell viability and IC50 values of KW (**A**), WQIWYK (**B**), and KGKWQIWYKSL (**C**) measured at various concentrations. Results are expressed as mean ± standard deviation from three independent experiments (n = 3).

To further investigate the anticancer activity of these three peptides, a concentration-dependent analysis was conducted to determine their IC50 values. As illustrated in Figure 7, the IC50 values were determined as follows: KW for A375 cells at 104.7 mg/mL, WQIWYK for K562 cells at 86.06 mg/mL, and KGKWQIWYKSL for HT-29 cells at 97.79 mg/mL. The IC50 value, defined as the concentration required to achieve a 50% inhibition rate, serves as a critical indicator of inhibitory efficacy. Lower IC50 values correlate with higher inhibitory potency, as they reflect the ability to achieve the desired effect at reduced concentrations. Among the tested peptides, WQIWYK exhibited the lowest IC50 value, followed by KGKWQIWYKSL and KW, suggesting that WQIWYK might possess the most potent anticancer activity.

### 2.7. Safety Evaluation of the Peptides

**Figure 8:**
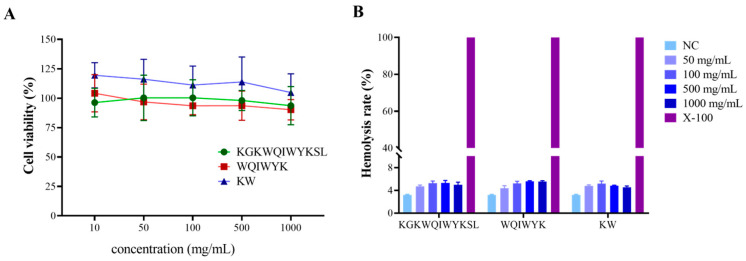
Biosafety evaluation of the peptide fragments. (**A**) Cytotoxicity of the peptides on 293FT cells; (**B**) hemolytic effects of KW, WQIWYK, and KGKWQIWYKSL, measured in three independent replicates (n = 3).

To further assess the safety profile of the three aforementioned peptides, cytotoxicity assays on normal cells and hemolysis experiments were conducted. The results demonstrated that the three peptide fragments exhibited no significant cytotoxicity toward normal cells at concentrations ranging from 10 to 1000 mg/mL (Figure 8A). Subsequently, hemolysis experiments were performed to evaluate the biocompatibility of these peptides. As shown in Figure 8B, the hemolysis rates of KW, WQIWYK, and KGKWQIWYKSL remained below 5% in a 2% (*v*/*v*) erythrocyte suspension, indicating minimal impact on erythrocyte membrane integrity. Given that systemic circulation represents the primary mode of drug distribution in vivo, the hemolytic potential of ACPs serves as a critical indicator of their biological safety and a key determinant in drug development. The peptide concentrations utilized in this study were confirmed to be within the safe range, thereby establishing a reliable foundation for the further development of ACPs.

## 3. Materials and Methods

### 3.1. Materials

The amino acids employed in the synthesis of peptides were procured from Kingsray Biotechnology Co., Ltd., Nanjing, China. 3-(4,5-Dimethylthiazol-2-yl)-2,5-diphenyltetrazolium bromide (MTT) was procured from Sigma Chemical Co. (St. Louis, MO, USA).

### 3.2. In Silico Evaluation of Arca-Derived Proteins as Precursors of Bioactive Peptides

Based on a comprehensive literature review, we identified four reported *Arca* proteins: three from *A. inflata* and one from *A. subcrenata*. To ensure the suitability of these proteins for further study, we applied the following inclusion criteria:

- The parent protein must have complete sequence information.
- Homology analysis should reveal low sequence identity (<80%).
- The initial protein must exhibit significant antitumor activity.

These criteria were designed to select proteins with diverse sequences and functional relevance, ensuring a robust foundation for subsequent analyses.

### 3.3. Simulated Proteolysis and Constructed the Peptide Library

Protein sequences were analyzed using the BIOPEP-UWM server (https://biochemia.uwm.edu.pl/en/biopep-uwm-2/, accessed on 16 March 2024) [62]. The “batch processing” function was employed to simulate enzymatic hydrolysis, cleaving peptide chains at enzyme-specific sites to generate peptide fragments. Five food-processing enzymes were selected:

- Chymotrypsin (EC 3.4.21.1);
- Trypsin (EC 3.4.21.4);
- Pepsin (pH 1.3, EC 3.4.23.1);
- Papain (EC 3.4.22.2);
- Subtilisin (EC 3.4.21.62).

The target protein sequences (ASP3, J2-C3) were input into the server, and the enzymes were selected for virtual hydrolysis. The resulting peptide fragments and hydrolysis degree data were collected. The DHt was calculated as the ratio of hydrolyzed peptide bonds to total peptide bonds. This approach was applicable to proteins with known amino acid sequences and enzymes with well-characterized cleavage specificities.

Additional details:

- All simulations were performed using default parameters provided by the BIOPEP-UWM server.
- The enzyme-to-substrate ratio was set to 1:100 (*w*/*w*), and the reaction time was standardized to 24 h for consistency.

### 3.4. Prediction of Peptides’ Solubility and Toxicity

Unreported peptide fragments were identified through comparative analysis with the BIOPEP-UWM database and existing literature. The ToxinPred platform (https://webs.iiitd.edu.in/raghava/toxinpred/index.html, accessed on 16 March 2024) [63] was used to evaluate the solubility and toxicological profiles of these peptides. These physicochemical properties were critical determinants of the biosafety and therapeutic potential of *Arca*-derived protein hydrolysates.

Additional details:

- ToxinPred was run using default parameters, including a threshold of 0.5 for toxicity prediction.
- Solubility predictions were based on the Kyte–Doolittle hydrophobicity scale, with peptides scoring below 0.5 considered highly soluble.

### 3.5. Prediction of Scores Indicating the Bioactive Potential for the Peptides

The PeptideRanker tool (http://distilldeep.ucd.ie/PeptideRanker/, accessed on 2 April 2024) was used to quantitatively assess the bioactive potential of peptide fragments. This bioinformatics analysis generates a probability score ranging from 0 to 1, with scores above 0.5 indicating a high likelihood of bioactivity.

Additional details:

- The PeptideRanker model was applied using default settings, including a threshold of 0.5 for bioactivity prediction.
- Peptides with scores >0.5 were prioritized for further analysis.

### 3.6. Prediction of Stability and Function for the Peptides

Peptide stability was evaluated using the ProtParam tool (https://web.expasy.org/protparam/, accessed on 2 April 2024). Stability was determined as a key parameter for ACPs, with instability indices calculated to predict potential degradation.

For peptides with molecular weights <1000 g/mol, structural formulas were drawn using ChemDraw 18.1 and saved in SMILES format. These structures were uploaded to the SwissTargetPrediction platform (http://swisstargetprediction.ch/, accessed on 10 April 2024), which outputs two types of data: (1) A pie chart (Figure 4) showing the statistical distribution of target classes. (2) Detailed target information, as provided in the Supplementary Tables. Targets identified by SwissTargetPrediction were cross-referenced with the Open Targets platform (https://platform.opentargets.org/, accessed on 10 April 2024) for therapeutic indication validation.

For peptides with molecular weights ≥1000 g/mol, amino acid sequences were input into the I-TASSER tool (https://zhanggroup.org/I-TASSER/, accessed on 10 April 2024) to generate 3D structural models (Figure 5) and predict potential functions (Table 2, Table 3, Table 4, Table 5 and Table 6). I-TASSER employs template identification via LOMETS meta-threading of PDB structures, followed by fragment reassembly and ab initio modeling, with structural refinement through SPICKER clustering and REMO optimization. Functional predictions were generated by comparing modeled structures against EC, GO, and ligand-binding databases, weighted by confidence scores, TM-scores, and sequence identity.

Additional details:

- SwissTargetPrediction was run using default parameters, including a probability threshold of 0.5 for target prediction.
- I-TASSER simulations were performed using a significance threshold (Z-score > 1.0) for template identification, a cutoff distance of 4.0 Å to identify low free-energy states, and the default C-score cutoff of −1.5 for model reliability.

### 3.7. Preparation of Peptides

The peptides (purity > 90%) were procured from Kingsray Biotechnology Co., Ltd. (Nanjing, China). The peptide synthesis was conducted through solid-phase methodology, followed by purification using reversed-phase high-performance liquid chromatography (RP-HPLC).

### 3.8. Cell Viability Assay

Cell lines were obtained from the Cell Bank of the Chinese Academy of Sciences (Shanghai, China) and maintained in Dulbecco’s Modified Eagle Medium (DMEM) or RPMI-1640 medium supplemented with 10% fetal bovine serum (FBS) at 37 °C in a humidified 5% CO_2_ atmosphere. For experimental analysis, cells were seeded at a density of 2 × 10^3^ cells/well in 96-well microplates and treated with 100 μg/mL of selected peptides for 48 h. The experimental panel included the following cell lines: colorectal carcinoma (HT-29, DLD-1, HCT-116), non-small-cell lung cancer (H460, HCC827, A549), lymphoma (Doudi, Farage), leukemia (K562, THP-1, HL-60), and other tumor cell lines (A375, MCF-7, Hela, HepG2). Cell viability was assessed using MTT assay, with 20 μL of MTT solution (5 mg/mL) added to each well, followed by measurement of optical density at 570 nm. Untreated cells served as negative controls. The IC50 values and safety evaluation procedures for KW, WQIWYK, and KGKWQIWYKSL were performed using the methods described previously.

### 3.9. Hemolysis Assay

The hemolytic activity was evaluated using a modified reference method. Briefly, sheep erythrocytes were isolated by centrifugation at 1000× *g* for 5 min following washing with physiological saline. The washing procedure was repeated until the supernatant became colorless, and the erythrocyte pellet was collected. A 2% (*v*/*v*) erythrocyte suspension was prepared in physiological saline. Aliquots (1 mL) of the suspension were incubated with peptide solutions (KW, WQIWYK, KGKWQIWYKSL) at concentrations of 0.05, 0.1, 0.5, and 1 mg/mL. Experimental controls included 0.1% Triton X-100 (positive control) and sterile PBS (negative control). Following incubation at 37 °C for 30 min, samples were centrifuged at 1000× *g* for 5 min. Hemoglobin release was quantified by measuring the absorbance of 200 μL supernatant at 425 nm using a microplate reader. The hemolysis percentage was calculated as follows:

Hemolysis (%) = (As − AN)/(AP − AN) × 100

where As, AN, and AP represent the absorbance values of the sample, negative control, and positive control, respectively.

### 3.10. Data Analysis

All experimental procedures were performed in triplicate, with data expressed as mean ± standard deviation. Statistical analyses were performed using GraphPad Prism^®^ software (version 8.00), employing one-way analysis of variance (ANOVA) with Newman–Keuls post hoc tests for multiple comparisons. *p* values < 0.05 were considered to be statistically significant.

## 4. Conclusions

This study presents an integrated computational–experimental framework for bioactive peptide screening, combining multiple bioinformatics tools (BIOPEP-UWM, PeptideRanker, ToxinPred, and ProtParam) with experimental validation. Traditional peptide purification relies on enzymatic hydrolysis coupled with chromatographic techniques (size-exclusion, ion-exchange, and reverse-phase chromatography). These methods exhibit three key limitations: prolonged processing times, suboptimal yields with significant byproduct generation, and substantial environmental burdens from solvent waste and resource intensity. In our study, the integrated bioinformatics pipeline addresses these challenges through four key analytical modules: (1) shortened experimental cycle—BIOPEP-UWM predicted proteolytic fragments, improved purification efficiency and reduced the generation of byproducts; (2) bioactivity prediction—PeptideRanker prioritizes candidates using a 0-1 bioactivity probability score (>0.5 considered bioactive); (3) safety screening—ToxinPred evaluates toxicity risks using a threshold of 0.5 for toxicity classification; and (4) stability analysis—ProtParam calculates instability indices (<40 considered stable).

While conventional methods focus on purity and quantitative analysis, our approach enhances characterization through physicochemical profiling (ProtParam); functional prediction (SwissTargetPrediction; confidence score > 0.5); and 3D structure modeling (I-TASSER; C-score > −1.5). Conventional activity screening methodologies predominantly rely on fluorescence-based assays or conjugated probe detection systems for target identification. Bioinformatics complements these methods by predicting active sites and binding affinities, thereby facilitating activity prediction.

However, it is important to acknowledge the limitations of bioinformatics approaches. Although in silico analyses offer important preliminary data, subsequent experimental verification remains crucial for confirming the predicted biological activities. The reliability of many tools and databases is contingent upon the quality and quantity of underlying data models. Despite these limitations, ongoing technological advancements are expected to address current challenges and enhance the predictive capabilities of bioinformatics in peptide research.

In conclusion, seventeen peptides were successfully identified from *Arca* species by using BIOPEP-UWM for simulated hydrolysis, PeptideRanker for activity index calculation, ToxinPred for toxicity prediction, ProtParam for stability evaluation, SwissTargetPrediction, and I-TASSER for target prediction. Then, through in vitro experiments, three new peptides (KW, WQIWYK, and KGKWQIWYKSL) were found to have relatively good anticancer activity, high biosafety, and potential for clinical application.

## Figures and Tables

**Table 1:** The amino acid sequence, PeptideRanker’s score, toxicity, hydropathicity, molecular weight, stability, sources, and enzymes used in processing of peptides, predicted by BIOPEP-UWM, PeptideRanker, ToxinPred, and ProtParam.

Peptide	PeptideRanker’s Score	Toxicity	Hydropathicity	Mol wt (g/mol)	Stability	Sources	Enzymes
QF	0.946135	non-toxic	−0.35	293.34	unknown	J2-C3	papain
KW	0.891359	non-toxic	−2.4	332.42	unknown	J2-C3	papain
WQIWYK	0.863206	non-toxic	−1	923.17	stable	J2-C3	trypsin
DSGF	0.809544	non-toxic	−0.48	424.45	unknown	J2-C3	Chymotrypsin /pepsin
DMESWWKKYVF	0.800725	non-toxic	−0.89	1518.9	stable	J2-C3	papain
KGKWQIWYKSL	0.762635	non-toxic	−1.02	1436.89	stable	J2-C3	pepsin
GDKASGVKVDMESWWNTYIF	0.750464	non-toxic	−0.43	2333.89	stable	ASP3	pepsin
GDKAQGVKTDMESWWKKYVF	0.737327	non-toxic	−1	2404.03	stable	J2-C3	pepsin
AFQLLNPK	0.673744	non-toxic	−0.04	930.23	stable	ASP3	trypsin
KGKW	0.661961	non-toxic	−2.27	517.68	unknown	J2-C3	Chymotrypsin /subtilisin
SKWKL	0.643756	non-toxic	−1.14	660.88	stable	ASP3	papain
NKF	0.623609	non-toxic	−1.53	407.5	unknown	J2-C3/ ASP3	papain
RNKF	0.606419	non-toxic	−2.28	563.7	unknown	J2-C3/ ASP3	subtilisin
MDY	0.59104	non-toxic	−0.97	427.5	unknown	J2-C3/ ASP3	subtilisin
VDMESWWNTYIFK	0.586957	non-toxic	−0.43	1719.13	stable	ASP3	trypsin
QIWYKSL	0.548062	non-toxic	−0.3	937.21	stable	J2-C3	papain
MEQF	0.527912	non-toxic	−0.57	553.68	unknown	J2-C3	subtilisin

PeptideRanker’s score is generated by PeptideRanker (range: 0–1), with higher scores indicating increased likelihood of bioactivity. Toxicity and hydropathicity are predicted using ToxinPred. Peptides identified as toxic may have potential safety concerns in clinical applications, while lower hydropathicity values correlate with improved water solubility. Stability was evaluated by the instability index (II) via ProtParam. Peptides with II ≥ 40 are classified as unstable and susceptible to degradation, whereas II < 40 indicates stable peptides with prolonged half-life in vivo.

**Table 2:** The binding, C-score, cluster size, PDB hit, ligand binding site residues, and target name for DMESWWKKYVF, predicted by the I-TASSER tool.

Binding	C-Score	Cluster Size	PDB Hit	Ligand Binding Site Residues	Target Name
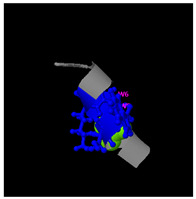	0.34	763	1vrgB	4, 5, 6, 7	Propionyl-CoA carboxylase
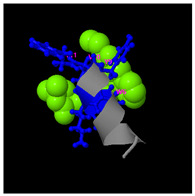	0.25	566	3tdcA	6, 7, 9, 10, 11	Acetyl-CoA carboxylase 2
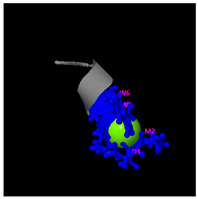	0.13	288	4eoyA	1, 2, 3, 4, 5, 6	Plasmodium falciparum Atg8 in complex with Plasmodium falciparum Atg3 peptide

C-score is the confidence score of the prediction. C-score ranges [0–1], where a higher score indicates a more reliable prediction. Cluster size is the total number of templates in a cluster. PDB hit is the combination of the PDB ID and a specific chain in the protein. Ligand binding site residues refers to the specific amino acid residues in proteins or other biological macromolecules that bind to ligands.

**Table 3:** The binding, C-score, cluster size, PDB hit, ligand binding site residues, and target name of VDMESWWNTYIFK, predicted by the I-TASSER tool.

Binding	C-Score	Cluster Size	PDB Hit	Ligand Binding Site Residues	Targets’ Name
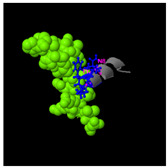	0.28	587	2zp9E	8, 9, 11, 12, 13	RNA Binding Protein
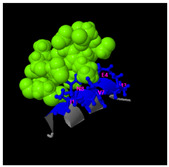	0.23	516	4ihgE	3, 4, 5, 7, 8, 11	Transferase/ lipid binding protein
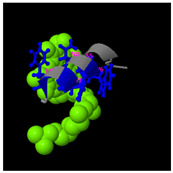	0.20	612	4pbui	5, 6, 8, 9, 10, 12	Enzymes involved in photosynthesis
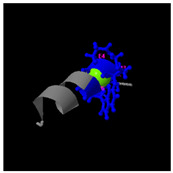	0.16	472	4eoyA	2, 3, 4, 5, 6	Transport proteases of Plasmodium falciparum Atg8 and Plasmodium falciparum Atg3

C-score is the confidence score of the prediction. C-score ranges [0–1], where a higher score indicates a more reliable prediction. Cluster size is the total number of templates in a cluster. PDB hit is the combination of the PDB ID and a specific chain in the protein. Ligand binding site residues refers to the specific amino acid residues in proteins or other biological macromolecules that bind to ligands.

**Table 4:** The binding, C-score, cluster size, PDB hit, ligand binding site residues and target name of KGKWQIWYKSL, predicted by the I-TASSER tool.

Binding	C-Score	Cluster Size	PDB Hit	Ligand Binding Site Residues	Target Name
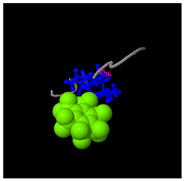	0.35	6	4cmxA	5, 6, 8	Nucleoprotein of Mycobacterium tuberculosis H37Rv
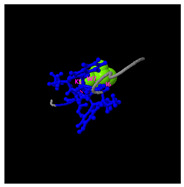	0.11	2	1cvnA	6, 7, 8, 9, 10	Lectin
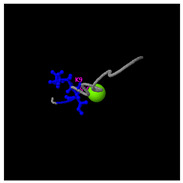	0.06	1	1jwyB	9, 10	Hydrolase of *Dictyostelium discoideum*

C-score is the confidence score of the prediction. C-score ranges [0–1], where a higher score indicates a more reliable prediction. Cluster size is the total number of templates in a cluster. PDB hit is the combination of the PDB ID and a specific chain in the protein. Ligand binding site residues refers to the specific amino acid residues in proteins or other biological macromolecules that bind to ligands.

**Table 5:** The binding, C-score, cluster size, PDB hit, ligand binding site residues and target name of GDKASGVKVDMESWWNTYIF, predicted by I-TASSER tool.

Binding	C-Score	Cluster Size	PDB Hit	Ligand Binding Site Residues	Target Name
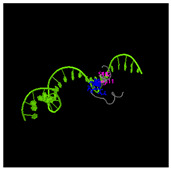	0.24	66	4awlC	10, 11, 12, 13	DNA Transcriptase (Human)
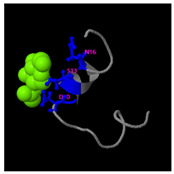	0.24	67	1r46B	10, 13, 16	α-Galactosidase
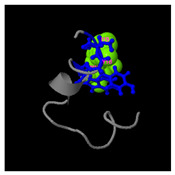	0.08	23	2wksB	10, 13, 16	Helicobacter pylori type II dehydroquinase
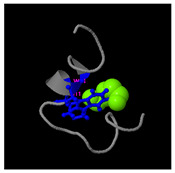	0.02	5	4gukC	11, 14	human NCS1 (Protein binding)
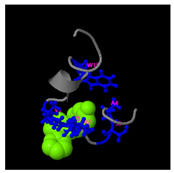	0.01	4	4nblB	4, 5, 7, 8, 9, 15	Procaspase-6

C-score is the confidence score of the prediction. C-score ranges [0–1], where a higher score indicates a more reliable prediction. Cluster size is the total number of templates in a cluster. PDB hit is the combination of the PDB ID and a specific chain in the protein. Ligand binding site residues refers to the specific amino acid residues in proteins or other biological macromolecules that bind to ligands.

**Table 6:** The binding, C-score, cluster size, PDB hit, ligand binding site residues and target name of GDKAQGVKTDMESWWKKYVF, predicted by the I-TASSER tool.

Binding	C-Score	Cluster Size	PDB Hit	Ligand Binding Site Residues	Target Name
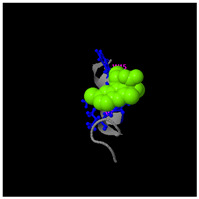	0.24	312	4dmnA	7, 8, 11, 12, 15	HIV-1 integrase
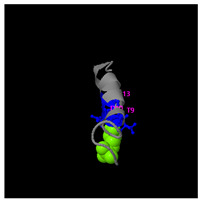	0.14	186	4rku4	9, 10, 13	Enzymes related to photosynthesis
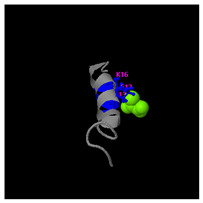	0.12	158	4iqdA	12, 13, 16	Carboxyvinyl-carboxyphosphonate phosphorylmutase
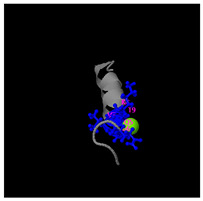	0.10	130	3i8gW	5, 6, 7, 8, 9	70S ribosome (eukaryotic organisms)
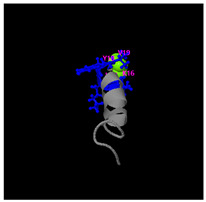	0.07	90	4id9A	16, 17, 18, 19	Short-chain dehydrogenase/ reductase superfamily proteins [Agrobacterium tumefaciens (TARGET EFI-506441)]

C-score is the confidence score of the prediction. C-score ranges [0–1], where a higher score indicates a more reliable prediction. Cluster size is the total number of templates in a cluster. PDB hit is the combination of the PDB ID and a specific chain in the protein. Ligand binding site residues refers to the specific amino acid residues in proteins or other biological macromolecules that bind to ligands.

## Data Availability

Data are contained within the article and in the Supplementary Materials.

## Supplementary Materials

The following supporting information can be downloaded at: https://www.mdpi.com/article/10.3390/molecules30071640/s1, Table S1. Prediction of potential targets of RNKF peptides. Table S2. Prediction of potential targets of KW peptides. Table S3. Prediction of potential targets of MEQF peptides. Table S4. Prediction of potential targets of QF peptides. Table S5. Prediction of potential targets of KGKW peptides. Table S6. Prediction of potential targets of NKF peptides. Table S7. Prediction of potential targets of DSGF peptides. Table S8. Prediction of potential targets of MDY peptides. Table S9. Prediction of potential targets of QIWYKSL peptides. Table S10. Prediction of potential targets of SKWKL peptides. Table S11. Prediction of potential targets of WQIWYK peptides. Table S12. Prediction of potential targets of AFQLLNPK peptides.
