# Supplementary material for: Investigation of Anticancer Peptides Derived from Arca Species Using In Silico Analysis
Source: Molecules. 2025 Apr 7;30(7):1640. doi: 10.3390/molecules30071640 (PMC11990805; doi:10.3390/molecules30071640)
Supplement: Supplementary file 1 [file molecules-30-01640-s001.zip › molecules-3530167-supplementary.pdf]

Investigation of Anti-cancer Peptides Derived From *Arca* Species Using *in Silico* Analysis

Jixu Wu [a,b,†], Xiuhua Zhang [b,†], Yuting Jin [a], Man Zhang [a], Rongmin Yu [a], Liyan Song [a], Fei Liu [b,] and Jianhua Zhu [a,*]**

[a] *Biotechnological Institute of Chinese Materia Medica, Jinan University, Guangzhou 510632, China*

[b] *Key Laboratory of Biopharmaceuticals, Engineering Laboratory of Polysaccharide Drugs, National-Local Joint Engineering Laboratory of Polysaccharide Drugs, Shandong Academy of Pharmaceutical Science,Jinan 250101, China*

[†] These authors contributed equally to this work and should be considered co-first authors.

* Corresponding author

**Corresponding author

E-mail addresses: tzhujh@jnu.edu.cn (J. Zhu); lfshwu@163.com (F. Liu).

Table S1. Prediction of potential targets of RNKF peptides

Target	Common name	Target Class	Probability*
CDK2/Cyclin A	CCNA2 CDK2	Kinase	0.215451689
Integrin alpha-V/beta-3	ITGAV ITGB3	Membrane receptor	0.169660852
Cyclin-dependent kinase 4/ cyclin D1	CCND1 CDK4	Kinase	0.142092181
Neurokinin 1 receptor (by homology)	TACR1	Family A G protein-coupled receptor	0.132852459
Integrin alpha-IIb/beta-3	ITGA2B ITGB3	Membrane receptor	0.123591725
Neurotensin receptor 1	NTSR1	Family A G protein-coupled receptor	0.114503358
Integrin alpha-5/beta-1	ITGB1 ITGA5	Membrane receptor	0.105306196
Integrin alpha-V/beta-6	ITGAV ITGB6	Membrane receptor	0.105306196
Acidic mammalian chitinase	CHIA	Enzyme	0.096101092
Delta opioid receptor (by homology)	OPRD1	Family A G protein-coupled receptor	0.096101092
Integrin alpha-V/beta-5	ITGB5 ITGAV	Membrane receptor	0.096101092
Mu opioid receptor	OPRM1	Family A G protein-coupled receptor	0.096101092
Neurotensin receptor 2	NTSR2	Family A G protein-coupled receptor	0.096101092
Thrombin	F2	Protease	0.096101092
Aminopeptidase N	ANPEP	Protease	0.086885855
Beta-secretase 1	BACE1	Protease	0.086885855
Histone deacetylase 3	HDAC3	Eraser	0.086885855
Histone deacetylase 6	HDAC6	Eraser	0.086885855
Neuromedin-U receptor 1	NMUR1	Family A G protein-coupled receptor	0.086885855
Neuromedin-U receptor 2	NMUR2	Family A G protein-coupled receptor	0.086885855
Neuropeptide Y receptor type 4	NPY4R	Family A G protein-coupled receptor	0.086885855
Angiotensin II receptor	AGTR2	Family A G protein-coupled receptor	0.086885855
Angiotensin-converting enzyme	ACE	Protease	0.086885855
Beta secretase 2	BACE2	Protease	0.086885855
C-X-C chemokine receptor type 4	CXCR4	Family A G protein-coupled receptor	0.086885855
C-X-C chemokine receptor type 7	ACKR3	Family A G protein-coupled receptor	0.086885855
C3a anaphylatoxin chemotactic receptor	C3AR1	Family A G protein-coupled receptor	0.086885855
Calcitonin gene-related peptide type 1 receptor	CALCRL	Family B G protein-coupled receptor	0.086885855

Table S2. Prediction of potential targets of KW peptides

Target	Common name	Target Class	Probability*
Angiotensin-converting enzyme	ACE	Protease	0.459956
Ephrin type-A receptor 2	EPHA2	Kinase	0.13697
Ephrin type-B receptor 2	EPHB2	Kinase	0.13697
Ephrin type-A receptor 5	EPHA5	Kinase	0.13697
Ephrin type-A receptor 4	EPHA4	Kinase	0.13697
Ephrin type-A receptor 8	EPHA8	Kinase	0.13697
Ephrin type-A receptor 6	EPHA6	Kinase	0.13697
Ephrin type-A receptor 7	EPHA7	Kinase	0.13697
Ephrin type-B receptor 3	EPHB3	Kinase	0.13697
Ephrin type-A receptor 3	EPHA3	Kinase	0.13697
Ephrin type-B receptor 1	EPHB1	Kinase	0.13697
Ephrin receptor	EPHB4	Kinase	0.13697
Ephrin type-A receptor 1	EPHA1	Kinase	0.13697
Ephrin receptor	EPHB6	Unclassified protein	0.13697
Neurotensin receptor 1 (by homology)	NTSR1	Family A G protein-coupled receptor	0.128899
Chromobox protein homolog 7	CBX7	Reader	0.128899
Somatostatin receptor 5	SSTR5	Family A G protein-coupled receptor	0.128899
Somatostatin receptor 2	SSTR2	Family A G protein-coupled receptor	0.128899
Somatostatin receptor 4	SSTR4	Family A G protein-coupled receptor	0.128899
Somatostatin receptor 1	SSTR1	Family A G protein-coupled receptor	0.120824
Somatostatin receptor 3	SSTR3	Family A G protein-coupled receptor	0.120824
Epoxide hydratase	EPHX2	Protease	0.120824
Neurokinin 1 receptor	TACR1	Family A G protein-coupled receptor	0.120824
Membrane-associated guanylate kinase-related 3	MAGI3	Enzyme	0.112748
HLA class I histocompatibility antigen A-3	HLA-A	Surface antigen	0.112748
T-cell protein-tyrosine phosphatase	PTPN2	Phosphatase	0.112748
Cathepsin L	CTSL	Protease	0.104672
Cathepsin (B and K)	CTSB	Protease	0.104672
Histone deacetylase 3	HDAC3	Eraser	0.104672
Histone deacetylase 6	HDAC6	Eraser	0.104672
Histone deacetylase 2	HDAC2	Eraser	0.104672
Endothelin receptor ET-A	EDNRA	Family A G protein-coupled receptor	0.104672
Neprilysin	MME	Protease	0.104672
Glucagon receptor	GCGR	Family B G protein-	0.104672

(by homology)		coupled receptor	
Endothelin receptor ET-B	EDNRB	Family A G protein-coupled receptor	0.104672
Mu opioid receptor (by homology)	OPRM1	Family A G protein-coupled receptor	0.104672
Vasopressin V2 receptor (by homology)	AVPR2	Family A G protein-coupled receptor	0.104672
Vasopressin V1a receptor (by homology)	AVPR1A	Family A G protein-coupled receptor	0.104672
Oxytocin receptor (by homology)	OXTR	Family A G protein-coupled receptor	0.104672
Melanocortin receptor 4	MC4R	Family A G protein-coupled receptor	0.104672
Melanocortin receptor 3	MC3R	Family A G protein-coupled receptor	0.104672
C5a anaphylatoxin chemotactic receptor	C5AR1	Family A G protein-coupled receptor	0.104672
CMP-N-acetylneuraminate-beta-galactosamide-alpha-2,3-sialyltransferase 1	ST3GAL1	Transferase	0.104672
Neurokinin 3 receptor	TACR3	Family A G protein-coupled receptor	0.104672
Cholecystokinin A receptor	CCKAR	Family A G protein-coupled receptor	0.104672
Histone deacetylase 3/Nuclear receptor corepressor 2 (HDAC3/NCoR2)	NCOR2 HDAC3	Eraser	0.104672
Histone deacetylase 8	HDAC8	Eraser	0.104672
Histone deacetylase 11	HDAC11	Eraser	0.104672
Histone deacetylase 10	HDAC10	Eraser	0.104672
Dipeptidyl peptidase IV	DPP4	Protease	0.104672
Cholecystokinin B receptor (by homology)	CCKBR	Family A G protein-coupled receptor	0.104672
Integrin alpha-IIb/beta-3	ITGA2B ITGB3	Membrane receptor	0.104672
Matrix metalloproteinase 1	MMP1	Protease	0.104672
Matrix metalloproteinase 7	MMP7	Protease	0.104672
Histone deacetylase 4	HDAC4	Eraser	0.104672
Ghrelin receptor	GHSR	Family A G protein-coupled receptor	0.104672
Neurokinin 2 receptor	TACR2	Family A G protein-coupled receptor	0.104672
Proteasome subunit beta type-9	PSMB9	Enzyme	0.104672
Proteasome subunit beta type-8	PSMB8	Protease	0.104672
Dipeptidyl peptidase I	CTSC	Protease	0.104672

Melanocortin receptor 5	MC5R	Family A G protein-coupled receptor	0.104672
Protein farnesyltransferase	FNTA FNTB	Enzyme	0.104672
G-protein coupled bile acid receptor 1	GPBAR1	Family A G protein-coupled receptor	0.104672
Cathepsin D	CTSD	Protease	0.104672
Matrix metalloproteinase 16	MMP16	Protease	0.104672
ADAMTS5	ADAMTS5	Protease	0.104672
Matrix metalloproteinase 17	MMP17	Protease	0.104672
Matrix metalloproteinase 15	MMP15	Protease	0.104672
ADAM17	ADAM17	Protease	0.104672
Matrix metalloproteinase 26	MMP26	Protease	0.104672
ADAM10	ADAM10	Protease	0.104672
ADAM12	ADAM12	Protease	0.104672
ADAM9	ADAM9	Protease	0.104672
Delta opioid receptor	OPRD1	Family A G protein-coupled receptor	0.104672
Nociceptin receptor	OPRL1	Family A G protein-coupled receptor	0.104672
Leukotriene A4 hydrolase	LTA4H	Protease	0.104672
Matrix metalloproteinase 2	MMP2	Protease	0.104672
Matrix metalloproteinase 8	MMP8	Protease	0.104672
Matrix metalloproteinase 9	MMP9	Protease	0.104672
Intercellular adhesion molecule (ICAM-1), Integrin alpha-L/beta-2	ITGAL ICAM1 ITGB2	Membrane receptor	0.104672
Baculoviral IAP repeat-containing protein 2	BIRC2	Enzyme	0.104672
Peroxisome proliferator-activated receptor gamma	PPARG	Nuclear receptor	0.104672
Protein-tyrosine phosphatase 1B	PTPN1	Phosphatase	0.104672
Endoplasmic reticulum aminopeptidase 2	ERAP2	Protease	0.104672
Cyclophilin A	PPIA	Isomerase	0.104672
Kappa Opioid receptor	OPRK1	Family A G protein-coupled receptor	0.104672
Neurotensin receptor 2	NTSR2	Family A G protein-coupled receptor	0.104672
Menin	MEN1	Unclassified protein	0.104672
Melatonin receptor 1A	MTNR1A	Family A G protein-coupled receptor	0.104672
Calpain 1	CAPN1	Protease	0.104672
Endothelin-converting enzyme 1	ECE1	Protease	0.104672
Cystinyl aminopeptidase	LNPEP	Protease	0.104672
Renin	REN	Protease	0.104672
Coagulation factor VII/ tissue factor	F3	Surface antigen	0.104672

Galanin receptor 1 (by homology)	GALR1	Family A G protein-coupled receptor	0.104672
Galanin receptor 2 (by homology)	GALR2	Family A G protein-coupled receptor	0.104672
Protein kinase C gamma	PRKCG	Kinase	0.104672
Protein kinase C alpha	PRKCA	Kinase	0.104672
Protein kinase C beta	PRKCB	Kinase	0.104672
Protein kinase C epsilon	PRKCE	Kinase	0.104672

Table S3. Prediction of potential targets of MEQF peptides

**Target**	**Common name**	**Target Class**	**Probability***
Beta-secretase 1	BACE1	Protease	0.311638
Formyl peptide receptor 1	FPR1	Family A G protein-coupled receptor	0.146653
HMG-CoA reductase	HMGCR	Oxidoreductase	0.130154
Mu opioid receptor (by homology)	OPRM1	Family A G protein-coupled receptor	0.130154
Disks large homolog 4	DLG4	Unclassified protein	0.130154
Cathepsin D	CTSD	Protease	0.121906
Neurokinin 3 receptor	TACR3	Family A G protein-coupled receptor	0.121906
Delta opioid receptor (by homology)	OPRD1	Family A G protein-coupled receptor	0.121906
Angiotensin-converting enzyme	ACE	Protease	0.121906
Inhibitor of apoptosis protein 3	XIAP	Other cytosolic protein	0.121906
Protein farnesyltransferase	FNTA FNTB	Enzyme	0.121906
HLA class I histocompatibility antigen A-3	HLA-A	Surface antigen	0.121906
Oxytocin receptor	OXTR	Family A G protein-coupled receptor	0.121906
C5a anaphylatoxin chemotactic receptor	C5AR1	Family A G protein-coupled receptor	0.121906
Neprilysin	MME	Protease	0.121906
Neurokinin 1 receptor (by homology)	TACR1	Family A G protein-coupled receptor	0.121906
Beta secretase 2	BACE2	Protease	0.121906
Aminopeptidase N	ANPEP	Protease	0.121906
Integrin alpha-4/beta-1	ITGB1 ITGA4	Membrane receptor	0.121906
Tyrosine-protein kinase LCK	LCK	Kinase	0.121906

Endothelin-converting enzyme 1	ECE1	Protease	0.121906
Renin	REN	Protease	0.121906
Cathepsin E	CTSE	Protease	0.121906
Vasopressin V1b receptor	AVPR1B	Family A G protein-coupled receptor	0.121906
Kallikrein 1	KLK1	Protease	0.121906
Tyrosine-protein kinase SRC	SRC	Kinase	0.121906
Integrin alpha-IIb/beta-3	ITGA2B ITGB3	Membrane receptor	0.121906
Cyclin-dependent kinase 4/cyclin D1	CCND1 CDK4	Kinase	0.121906
CDK2/Cyclin A	CCNA2 CDK2	Kinase	0.121906
Geranylgeranyl transferase type I	PGGT1B FNTA	Enzyme	0.121906
Integrin alpha-V/beta-3	ITGAV ITGB3	Membrane receptor	0.121906
Neuropeptide FF receptor 1	NPFFR1	Family A G protein-coupled receptor	0.121906
Neuropeptide FF receptor 2	NPFFR2	Family A G protein-coupled receptor	0.121906
Calcitonin gene-related peptide type 1 receptor	CALCRL	Family B G protein-coupled receptor	0.121906
Squalene synthetase	FDFT1	Enzyme	0.121906
Prolactin receptor	PRLR	Unclassified protein	0.121906
Thrombin	F2	Protease	0.121906
Vasopressin V2 receptor	AVPR2	Family A G protein-coupled receptor	0.121906
Vasopressin V1a receptor	AVPR1A	Family A G protein-coupled receptor	0.121906
Serine/threonine-protein kinase Aurora-A	AURKA	Kinase	0.121906
Integrin alpha-2/beta-3	ITGA2 ITGB3	Membrane receptor	0.121906
C3a anaphylatoxin chemotactic receptor	C3AR1	Family A G protein-coupled receptor	0.121906
Thymidylate synthase (by homology)	TYMS	Transferase	0.121906
Beta-1 adrenergic receptor	ADRB1	Family A G protein-coupled receptor	0.121906
Cyclooxygenase-2	PTGS2	Oxidoreductase	0.121906
DNA (cytosine-5)-methyltransferase 1	DNMT1	Writer	0.121906
DNA (cytosine-5)-methyltransferase 3B	DNMT3B	Reader	0.121906
Chromobox protein homolog 7	CBX7	Reader	0.121906

E3 SUMO-protein ligase CBX4	CBX4	Enzyme	0.121906
Matrix metalloproteinase 3	MMP3	Protease	0.121906
Calcium sensing receptor	CASR	Family C G protein-coupled receptor	0.121906
Matrix metalloproteinase 1	MMP1	Protease	0.121906
Proenkephalin B	PDYN	Other ion channel	0.121906
Tyrosine-protein kinase SYK	SYK	Kinase	0.121906
Matrix metalloproteinase 9	MMP9	Protease	0.121906
Glutamate receptor ionotropic kainate 1	GRIK1	Ligand-gated ion channel	0.121906
Glutamate receptor ionotropic kainate 3	GRIK3	Ligand-gated ion channel	0.121906
Leucine aminopeptidase	LAP3	Protease	0.121906
Glutathione S-transferase Pi	GSTP1	Enzyme	0.121906
Histone-lysine N-methyltransferase, H3 lysine-79 specific	DOT1L	Writer	0.121906
Kappa Opioid receptor	OPRK1	Family A G protein-coupled receptor	0.121906
Uracil-DNA glycosylase	UNG	Enzyme	0.121906
Endoplasmic reticulum aminopeptidase 2	ERAP2	Protease	0.121906
Epoxide hydratase	EPHX2	Protease	0.121906
Endothelin receptor ET-A	EDNRA	Family A G protein-coupled receptor	0.121906
Serine/threonine protein phosphatase PP1-alpha catalytic subunit	PPP1CA	Phosphatase	0.121906
C-X-C chemokine receptor type 4	CXCR4	Family A G protein-coupled receptor	0.121906
Folate receptor alpha	FOLR1	Membrane receptor	0.121906
GAR transformylase	GART	Ligase	0.121906
Thromboxane-A synthase	TBXAS1	Cytochrome P450	0.121906
Carboxypeptidase A1	CPA1	Protease	0.121906
Glycogen synthase kinase-3 beta	GSK3B	Kinase	0.121906
Matrix metalloproteinase 2	MMP2	Protease	0.121906
Matrix metalloproteinase 12	MMP12	Protease	0.121906
Matrix metalloproteinase 8	MMP8	Protease	0.121906
Caspase-1	CASP1	Protease	0.121906
Dual specificity mitogen-activated protein kinase kinase 7	MAP2K7	Kinase	0.121906

Neurotensin receptor 3	SORT1	Membrane receptor	0.121906
Tyrosine-protein kinase JAK1	JAK1	Kinase	0.121906
Tyrosine-protein kinase JAK2	JAK2	Kinase	0.121906
Tubulin beta-1 chain	TUBB1	Structural protein	0.121906
Purinergic receptor P2Y12	P2RY12	Family A G protein-coupled receptor	0.121906
NAD-dependent deacetylase sirtuin 1	SIRT1	Eraser	0.121906
Prostanoid FP receptor	PTGFR	Family A G protein-coupled receptor	0.121906
Xaa-Pro aminopeptidase 1	XPNPEP1	Protease	0.121906
Xaa-Pro aminopeptidase 2	XPNPEP2	Protease	0.121906

Table S4. Prediction of potential targets of QF peptides

**Target**	**Common name**	**Target Class**	**Probability***
Angiotensin-converting enzyme	ACE	Protease	0.237885
Calpain 1	CAPN1	Protease	0.22133
Tyrosyl-tRNA synthetase	YARS	Enzyme	0.147257
Delta opioid receptor	OPRD1	Family A G protein-coupled receptor	0.114338
Cyclooxygenase-2	PTGS2	Oxidoreductase	0.114338
Neprilysin	MME	Protease	0.114338
Integrin alpha-V/beta-3	ITGAV ITGB3	Membrane receptor	0.1061
Integrin alpha-4/beta-1	ITGB1 ITGA4	Membrane receptor	0.1061
Aminopeptidase N	ANPEP	Protease	0.1061
Leucine aminopeptidase	LAP3	Protease	0.1061
Inhibitor of apoptosis protein 3	XIAP	Other cytosolic protein	0.1061
HLA class I histocompatibility antigen A-3	HLA-A	Surface antigen	0.1061
Mu opioid receptor	OPRM1	Family A G protein-coupled receptor	0.1061
Protein farnesyltransferase	FNTA FNTB	Enzyme	0.1061
Oligopeptide transporter small intestine isoform	SLC15A1	Electrochemical transporter	0.097875
Neurokinin 1 receptor (by homology)	TACR1	Family A G protein-coupled receptor	0.097875
Nitric oxide synthase, inducible	NOS2	Enzyme	0.097875
Cyclin-dependent kinase 4/cyclin D1	CCND1 CDK4	Kinase	0.097875

CDK2/Cyclin A	CCNA2 CDK2	Kinase	0.097875
Neurotensin receptor 1 (by homology)	NTSR1	Family A G protein-coupled receptor	0.097875
Leukotriene A4 hydrolase	LTA4H	Protease	0.097875
HMG-CoA reductase	HMGCR	Oxidoreductase	0.097875
Matrix metalloproteinase 2	MMP2	Protease	0.097875
Tyrosine-protein kinase SRC	SRC	Kinase	0.097875
Aminopeptidase B (by homology)	RNPEP	Protease	0.097875
Formyl peptide receptor 1	FPR1	Family A G protein-coupled receptor	0.097875
Beta-secretase 1	BACE1	Protease	0.097875
Cyclin-dependent kinase 2/cyclin A	CDK2 CCNA1 CCNA2	Other cytosolic protein	0.097875
Chromobox protein homolog 7	CBX7	Reader	0.097875
E3 SUMO-protein ligase CBX4	CBX4	Enzyme	0.097875
Integrin alpha-IIb/beta-3	ITGA2B ITGB3	Membrane receptor	0.097875
Neuropeptide FF receptor 1	NPFFR1	Family A G protein-coupled receptor	0.097875
Neuropeptide FF receptor 2	NPFFR2	Family A G protein-coupled receptor	0.097875
Kappa Opioid receptor	OPRK1	Family A G protein-coupled receptor	0.097875
Neurotensin receptor 2	NTSR2	Family A G protein-coupled receptor	0.097875
Calcitonin gene-related peptide type 1 receptor	CALCRL	Family B G protein-coupled receptor	0.097875
Dopamine transporter (by homology)	SLC6A3	Electrochemical transporter	0.097875
Signal transducer and activator of transcription 3	STAT3	Transcription factor	0.097875
Dipeptidyl peptidase IV	DPP4	Protease	0.097875
Proenkephalin B	PDYN	Other ion channel	0.097875
Epoxide hydratase	EPHX2	Protease	0.097875
Beta secretase 2	BACE2	Protease	0.097875
Thyrotropin-releasing hormone receptor (by homology)	TRHR	Family A G protein-coupled receptor	0.097875
NAD-dependent deacetylase sirtuin 2	SIRT2	Eraser	0.097875
Renin	REN	Protease	0.097875
Bradykinin B1 receptor	BDKRB1	Family A G protein-	0.097875

		coupled receptor	
Ribonucleoside-diphosphate reductase M1 chain (by homology)	RRM1	Oxidoreductase	0.097875
Neurokinin 2 receptor	TACR2	Family A G protein-coupled receptor	0.097875
Dipeptidyl peptidase VIII	DPP8	Protease	0.097875
Integrin alpha-5/beta-1	ITGB1 ITGA5	Membrane receptor	0.097875
Carboxypeptidase B2 isoform A	CPB2	Protease	0.097875
Kallikrein 1	KLK1	Protease	0.097875
Baculoviral IAP repeat-containing protein 3	BIRC3	Enzyme	0.097875
Baculoviral IAP repeat-containing protein 2	BIRC2	Enzyme	0.097875
Angiotensin-converting enzyme 2	ACE2	Protease	0.097875
Vasopressin V1a receptor (by homology)	AVPR1A	Family A G protein-coupled receptor	0.097875
Oxytocin receptor (by homology)	OXTR	Family A G protein-coupled receptor	0.097875
MAP kinase-activated protein kinase 2	MAPKAPK2	Kinase	0.097875
Xaa-Pro dipeptidase	PEPD	Protease	0.097875
Xaa-Pro aminopeptidase 2	XPNPEP2	Protease	0.097875
Sigma opioid receptor	SIGMAR1	Membrane receptor	0.097875
Thrombin	F2	Protease	0.097875
Disks large homolog 4	DLG4	Unclassified protein	0.097875
Dipeptidyl peptidase IX	DPP9	Protease	0.097875
Endothelin receptor ET-A	EDNRA	Family A G protein-coupled receptor	0.097875
Pyroglutamylated RFamide peptide receptor	QRFPR	Family A G protein-coupled receptor	0.097875
Sphingosine kinase 1	SPHK1	Enzyme	0.097875
Atrial natriuretic factor	NPPA	Unclassified protein	0.097875
HLA class II histocompatibility antigen DRB1-1	HLA-DRB1	Surface antigen	0.097875
Galanin receptor 1 (by homology)	GALR1	Family A G protein-coupled receptor	0.097875
Neurotensin receptor 3	SORT1	Membrane receptor	0.097875
Gamma-secretase	PSEN2 PSENEN NCSTN APH1A PSEN1 APH1B	Protease	0.097875
Cholecystokinin B receptor	CCKBR	Family A G protein-	0.097875

		coupled receptor	
Xaa-Pro aminopeptidase 1	XPNPEP1	Protease	0.097875
C-X-C chemokine receptor type 7	ACKR3	Family A G protein-coupled receptor	0.097875
Endothelin-converting enzyme 1	ECE1	Protease	0.097875
Max-like protein X	MLX	Unclassified protein	0.097875
Furin	FURIN	Protease	0.097875
Lysine-specific demethylase 4A	KDM4A	Eraser	0.097875
Lysine-specific demethylase 4C	KDM4C	Eraser	0.097875
Vasopressin V2 receptor (by homology)	AVPR2	Family A G protein-coupled receptor	0.097875
Cathepsin D	CTSD	Protease	0.097875
Cathepsin E	CTSE	Protease	0.097875
Alkaline ceramidase 2	ACER2	Enzyme	0.097875
Acid ceramidase	ASAH1	Enzyme	0.097875
Cyclin-dependent kinase 2	CDK2	Kinase	0.097875
Ghrelin receptor	GHSR	Family A G protein-coupled receptor	0.097875
Peptidyl-glycine alpha-amidating monooxygenase	PAM	Enzyme	0.097875
Mas-related G-protein coupled receptor member X1 (by homology)	MRGPRX1	Unclassified protein	0.097875
Ephrin type-A receptor 2	EPHA2	Kinase	0.097875
MAP kinase ERK2	MAPK1	Kinase	0.097875
Appetite-regulating hormone	GHRL	Unclassified protein	0.097875
HLA class II histocompatibility antigen DRB3-1	HLA-DRB3	Surface antigen	0.097875
Hydroxycarboxylic acid receptor 2	HCAR2	Family A G protein-coupled receptor	0.097875
Melanocortin receptor 4	MC4R	Family A G protein-coupled receptor	0.097875
Melanocortin receptor 3	MC3R	Family A G protein-coupled receptor	0.097875
Cyclophilin A	PPIA	Isomerase	0.097875
Serine/threonine-protein kinase MST2	STK3	Kinase	0.097875

Serine/threonine-protein kinase Aurora-A	AURKA	Kinase	0.097875
Serine/threonine-protein kinase PIM1	PIM1	Kinase	0.097875

Table S5. Prediction of potential targets of KGKW peptides

Target	Common name	Target Class	Probability*
Endothelin receptor ET-A	EDNRA	Family A G protein-coupled receptor	0.290705254
Endothelin receptor ET-B	EDNRB	Family A G protein-coupled receptor	0.282545282
Chromobox protein homolog 7	CBX7	Reader	0.241185858
Somatostatin receptor 5	SSTR5	Family A G protein-coupled receptor	0.233030976
Somatostatin receptor 1	SSTR1	Family A G protein-coupled receptor	0.216512701
Somatostatin receptor 3	SSTR3	Family A G protein-coupled receptor	0.216512701
Neurotensin receptor 1	NTSR1	Family A G protein-coupled receptor	0.200014776
Epoxide hydratase	EPHX2	Protease	0.183481385
Mu opioid receptor (by homology)	OPRM1	Family A G protein-coupled receptor	0.17523513
Membrane-associated guanylate kinase-related 3	MAGI3	Enzyme	0.166953109
Histone deacetylase 3	HDAC3	Eraser	0.150460996
Histone deacetylase 6	HDAC6	Eraser	0.150460996
Histone deacetylase 2	HDAC2	Eraser	0.150460996
Histone deacetylase 1	HDAC1	Eraser	0.150460996
Angiotensin-converting enzyme	ACE	Protease	0.150460996
Calpain 1	CAPN1	Protease	0.142209035
Cyclophilin A	PPIA	Isomerase	0.142209035
Ephrin type-A receptor 2	EPHA2	Kinase	0.142209035
Ephrin type-B receptor 2	EPHB2	Kinase	0.142209035
Ephrin type-A receptor 5	EPHA5	Kinase	0.142209035
Ephrin type-A receptor 4	EPHA4	Kinase	0.142209035
Ephrin type-A receptor 8	EPHA8	Kinase	0.142209035
Ephrin type-A receptor 6	EPHA6	Kinase	0.142209035
Ephrin type-A receptor 7	EPHA7	Kinase	0.142209035
Ephrin type-B receptor 3	EPHB3	Kinase	0.142209035
Ephrin type-A receptor 3	EPHA3	Kinase	0.142209035
Ephrin type-B receptor 1	EPHB1	Kinase	0.142209035
Ephrin receptor	EPHB4	Kinase	0.142209035

Ephrin type-A receptor 1	EPHA1	Kinase	0.142209035
Ephrin receptor	EPHB6	Unclassified protein	0.142209035
Somatostatin receptor 2	SSTR2	Family A G protein-coupled receptor	0.142209035
Vasopressin V2 receptor (by homology)	AVPR2	Family A G protein-coupled receptor	0.142209035
Vasopressin V1a receptor (by homology)	AVPR1A	Family A G protein-coupled receptor	0.142209035
Oxytocin receptor (by homology)	OXTR	Family A G protein-coupled receptor	0.142209035
Melanocortin receptor 3	MC3R	Family A G protein-coupled receptor	0.142209035
Glucagon receptor (by homology)	GCGR	Family B G protein-coupled receptor	0.142209035
C5a anaphylatoxin chemotactic receptor	C5AR1	Family A G protein-coupled receptor	0.142209035
Cholecystokinin B receptor	CCKBR	Family A G protein-coupled receptor	0.142209035
Ghrelin receptor	GHSR	Family A G protein-coupled receptor	0.133944629
Somatostatin receptor 4	SSTR4	Family A G protein-coupled receptor	0.133944629
Histone deacetylase 3/Nuclear receptor corepressor 2 (HDAC3/NCoR2)	NCOR2 HDAC3	Eraser	0.133944629
Histone deacetylase 8	HDAC8	Eraser	0.133944629
Histone deacetylase 11	HDAC11	Eraser	0.133944629
Histone deacetylase 10	HDAC10	Eraser	0.133944629
Neurokinin 3 receptor	TACR3	Family A G protein-coupled receptor	0.133944629
Neurokinin 1 receptor	TACR1	Family A G protein-coupled receptor	0.133944629
CMP-N-acetylneuraminate-beta-galactosamide-alpha-2,3-sialyltransferase 1	ST3GAL1	Transferase	0.133944629
Neprilysin	MME	Protease	0.133944629
Delta opioid receptor	OPRD1	Family A G protein-coupled receptor	0.133944629
Histone deacetylase 4	HDAC4	Eraser	0.125687253
Neurokinin 2 receptor	TACR2	Family A G protein-coupled receptor	0.125687253
HLA class I histocompatibility antigen A-3	HLA-A	Surface antigen	0.125687253
Kappa Opioid receptor	OPRK1	Family A G protein-coupled receptor	0.125687253

Matrix metalloproteinase 1	MMP1	Protease	0.125687253
Proteasome subunit beta type-9	PSMB9	Enzyme	0.125687253
Proteasome subunit beta type-8	PSMB8	Protease	0.125687253
T-cell protein-tyrosine phosphatase	PTPN2	Phosphatase	0.125687253
Cathepsin L	CTSL	Protease	0.125687253
Cathepsin (B and K)	CTSB	Protease	0.125687253
Melanocortin receptor 5	MC5R	Family A G protein-coupled receptor	0.125687253
Matrix metalloproteinase 7	MMP7	Protease	0.125687253
Matrix metalloproteinase 2	MMP2	Protease	0.125687253
C3a anaphylatoxin chemotactic receptor	C3AR1	Family A G protein-coupled receptor	0.125687253
Nociceptin receptor	OPRL1	Family A G protein-coupled receptor	0.125687253
Melanocortin receptor 1	MC1R	Family A G protein-coupled receptor	0.125687253
Matrix metalloproteinase 8	MMP8	Protease	0.125687253
Baculoviral IAP repeat-containing protein 2	BIRC2	Enzyme	0.125687253
Cholecystokinin A receptor	CCKAR	Family A G protein-coupled receptor	0.125687253
Melanocortin receptor 4	MC4R	Family A G protein-coupled receptor	0.125687253
Renin	REN	Protease	0.125687253
Protein farnesyltransferase	FNTA FNTB	Enzyme	0.125687253
Menin	MEN1	Unclassified protein	0.125687253
Plasminogen	PLG	Protease	0.125687253
Matrix metalloproteinase 16	MMP16	Protease	0.125687253
ADAMTS5	ADAMTS5	Protease	0.125687253
Matrix metalloproteinase 17	MMP17	Protease	0.125687253
Matrix metalloproteinase 15	MMP15	Protease	0.125687253
ADAM17	ADAM17	Protease	0.125687253
Matrix metalloproteinase 14	MMP14	Protease	0.125687253
Matrix metalloproteinase 26	MMP26	Protease	0.125687253
ADAM10	ADAM10	Protease	0.125687253
ADAM12	ADAM12	Protease	0.125687253
ADAM9	ADAM9	Protease	0.125687253
Cathepsin D	CTSD	Protease	0.125687253
Neuropilin-1 (by homology)	NRP1	Secreted protein	0.125687253
Matrix metalloproteinase 3	MMP3	Protease	0.125687253
Inhibitor of apoptosis protein 3	XIAP	Other cytosolic protein	0.125687253

Galanin receptor 1 (by homology)	GALR1	Family A G protein-coupled receptor	0.125687253
Galanin receptor 2 (by homology)	GALR2	Family A G protein-coupled receptor	0.125687253
Leukotriene A4 hydrolase	LTA4H	Protease	0.125687253
Motilin receptor	MLNR	Family A G protein-coupled receptor	0.125687253
Serine/threonine-protein kinase PLK1	PLK1	Kinase	0.125687253
HLA class II histocompatibility antigen DRB1-1	HLA-DRB1	Surface antigen	0.125687253
Intercellular adhesion molecule (ICAM-1), Integrin alpha-L/beta-2	ITGAL ICAM1 ITGB2	Membrane receptor	0.125687253
Serotonin transporter (by homology)	SLC6A4	Electrochemical transporter	0.125687253
Matrix metalloproteinase 9	MMP9	Protease	0.125687253
Neurotensin receptor 2	NTSR2	Family A G protein-coupled receptor	0.125687253
Peroxisome proliferator-activated receptor gamma	PPARG	Nuclear receptor	0.125687253
Dipeptidyl peptidase I	CTSC	Protease	0.125687253
Endoplasmic reticulum aminopeptidase 2	ERAP2	Protease	0.125687253

Table S6. Prediction of potential targets of NKF peptides

Target	Common name	Target Class	Probability*
Inhibitor of apoptosis protein 3	XIAP	Other cytosolic protein	0.174623781
Angiotensin-converting enzyme	ACE	Protease	0.148309681
Cathepsin E	CTSE	Protease	0.121986395
Beta-secretase 1	BACE1	Protease	0.113206575
Delta opioid receptor	OPRD1	Family A G protein-coupled receptor	0.113206575
Mu opioid receptor	OPRM1	Family A G protein-coupled receptor	0.113206575
Cyclooxygenase-2	PTGS2	Oxidoreductase	0.104440651
Neurokinin 2 receptor	TACR2	Family A G protein-coupled receptor	0.104440651
Renin	REN	Protease	0.104440651
Thrombin	F2	Protease	0.104440651
Chromobox protein homolog 7	CBX7	Reader	0.104440651

E3 SUMO-protein ligase CBX4	CBX4	Enzyme	0.104440651
Integrin alpha-IIb/beta-3	ITGA2B ITGB3	Membrane receptor	0.095663487
Cathepsin D	CTSD	Protease	0.095663487
HLA class I histocompatibility antigen A-3	HLA-A	Surface antigen	0.095663487
Kappa Opioid receptor	OPRK1	Family A G protein-coupled receptor	0.095663487
Protein farnesyltransferase	FNTA FNTB	Enzyme	0.095663487
Neurotensin receptor 2	NTSR2	Family A G protein-coupled receptor	0.095663487
Beta secretase 2	BACE2	Protease	0.095663487
Integrin alpha-4	ITGA4	Membrane receptor	0.095663487
Integrin alpha-4/beta-1	ITGB1 ITGA4	Membrane receptor	0.095663487

Table S7. Prediction of potential targets of DSGF peptides

Target	Common name	Target Class	Probability*
Ribonucleoside-diphosphate reductase M1 chain (by homology)	RRM1	Oxidoreductase	0.131156
Neurokinin 2 receptor	TACR2	Family A G protein-coupled receptor	0.131156
Mu opioid receptor	OPRM1	Family A G protein-coupled receptor	0.122827
Calcitonin gene-related peptide type 1 receptor	CALCRL	Family B G protein-coupled receptor	0.122827
Beta-secretase 1	BACE1	Protease	0.122827
HMG-CoA reductase	HMGCR	Oxidoreductase	0.114495
Disks large homolog 4	DLG4	Unclassified protein	0.114495
Delta opioid receptor	OPRD1	Family A G protein-coupled receptor	0.106166
Integrin alpha-IIb/beta-3	ITGA2B ITGB3	Membrane receptor	0.106166
Beta secretase 2	BACE2	Protease	0.106166
HLA class I histocompatibility antigen A-3	HLA-A	Surface antigen	0.106166
Inhibitor of apoptosis protein 3	XIAP	Other cytosolic protein	0.106166
Cathepsin E	CTSE	Protease	0.106166
Integrin alpha-4/beta-1	ITGB1 ITGA4	Membrane receptor	0.106166
Renin	REN	Protease	0.106166
Integrin alpha-V/beta-3	ITGAV ITGB3	Membrane receptor	0.106166
Angiotensin-converting enzyme (by homology)	ACE	Protease	0.106166

Galanin receptor 1 (by homology)	GALR1	Family A G protein-coupled receptor	0.106166
Galanin receptor 2 (by homology)	GALR2	Family A G protein-coupled receptor	0.106166
Caspase-1	CASP1	Protease	0.106166
Leucine aminopeptidase	LAP3	Protease	0.106166
Neprilysin (by homology)	MME	Protease	0.106166
HLA class II histocompatibility antigen DRB3-1	HLA-DRB3	Surface antigen	0.106166
Tyrosyl-tRNA synthetase	YARS	Enzyme	0.106166
Chromobox protein homolog 7	CBX7	Reader	0.106166
E3 SUMO-protein ligase CBX4	CBX4	Enzyme	0.106166
Cholecystokinin B receptor	CCKBR	Family A G protein-coupled receptor	0.106166
Neuropeptide FF receptor 1	NPFFR1	Family A G protein-coupled receptor	0.106166
Neuropeptide FF receptor 2	NPFFR2	Family A G protein-coupled receptor	0.106166
Interleukin-1 beta	IL1B	Secreted protein	0.106166
Proenkephalin B	PDYN	Other ion channel	0.106166
Cyclooxygenase-2	PTGS2	Oxidoreductase	0.106166
Calpain 1	CAPN1	Protease	0.106166
Endothelin receptor ET-A	EDNRA	Family A G protein-coupled receptor	0.106166
Oxytocin receptor (by homology)	OXTR	Family A G protein-coupled receptor	0.106166
Formyl peptide receptor 1	FPR1	Family A G protein-coupled receptor	0.106166
Signal transducer and activator of transcription 3	STAT3	Transcription factor	0.106166
Angiotensin II receptor	AGTR2	Family A G protein-coupled receptor	0.106166
Tubulin beta-1 chain	TUBB1	Structural protein	0.106166
Neurokinin 1 receptor (by homology)	TACR1	Family A G protein-coupled receptor	0.106166
Matrix metalloproteinase 3	MMP3	Protease	0.106166
Matrix metalloproteinase 1	MMP1	Protease	0.106166
Bradykinin B1 receptor	BDKRB1	Family A G protein-coupled receptor	0.106166
ADAM17	ADAM17	Protease	0.106166
Cyclin-dependent kinase 4/cyclin D1	CCND1 CDK4	Kinase	0.106166
CDK2/Cyclin A	CCNA2 CDK2	Kinase	0.106166

Dopamine transporter (by homology)	SLC6A3	Electrochemical transporter	0.106166
Kappa Opioid receptor	OPRK1	Family A G protein-coupled receptor	0.106166
Cathepsin D	CTSD	Protease	0.106166
MAP kinase ERK2	MAPK1	Kinase	0.106166
Neurotensin receptor 1 (by homology)	NTSR1	Family A G protein-coupled receptor	0.106166
Vitronectin receptor alpha	ITGAV	Membrane receptor	0.106166
Lysine-specific histone demethylase 1	KDM1A	Eraser	0.106166
Muscarinic acetylcholine receptor M4	CHRM4	Family A G protein-coupled receptor	0.106166
Muscarinic acetylcholine receptor M2	CHRM2	Family A G protein-coupled receptor	0.106166
Muscarinic acetylcholine receptor M1	CHRM1	Family A G protein-coupled receptor	0.106166
Muscarinic acetylcholine receptor M3	CHRM3	Family A G protein-coupled receptor	0.106166
HLA class II histocompatibility antigen DRB1-1	HLA-DRB1	Surface antigen	0.106166
Neurokinin 3 receptor	TACR3	Family A G protein-coupled receptor	0.106166
Epoxide hydratase	EPHX2	Protease	0.106166
Histone-lysine N-methyltransferase, H3 lysine-79 specific	DOT1L	Writer	0.106166
DNA (cytosine-5)-methyltransferase 3B	DNMT3B	Reader	0.106166
Pyroglutamylated RFamide peptide receptor	QRFPR	Family A G protein-coupled receptor	0.106166
Cathepsin G	CTSG	Protease	0.106166
Beta-chymotrypsin	CTRB1	Protease	0.106166
Galectin-3	LGALS3	Other cytosolic protein	0.106166
Cyclin-dependent kinase 2/cyclin A	CDK2 CCNA1 CCNA2	Other cytosolic protein	0.106166
Integrin alpha-5/beta-1	ITGB1 ITGA5	Membrane receptor	0.106166
Kallikrein 1	KLK1	Protease	0.106166
Neurotensin receptor 2	NTSR2	Family A G protein-coupled receptor	0.106166
Aminopeptidase N	ANPEP	Protease	0.106166
Prolactin receptor	PRLR	Unclassified protein	0.106166
DNA (cytosine-5)-methyltransferase 1	DNMT1	Writer	0.106166

Tyrosine-protein kinase SRC	SRC	Kinase	0.106166
Histone-lysine N-methyltransferase SUV39H1	SUV39H1	Writer	0.106166
Indolethylamine N-methyltransferase	INMT	Enzyme	0.106166
EZH2/SUZ12/EED/RBBP7/RBBP4	EZH2	Writer	0.106166
Histone-lysine N-methyltransferase EZH1	EZH1	Writer	0.106166
Glyoxalase II	HAGH	Enzyme	0.106166
Histone-lysine N-methyltransferase SETDB1	SETDB1	Writer	0.106166
Histone-arginine methyltransferase CARM1	CARM1	Writer	0.106166
Protein-arginine N-methyltransferase 1	PRMT1	Writer	0.106166
Histone-lysine N-methyltransferase, H3 lysine-9 specific 5	EHMT1	Writer	0.106166
Histone-lysine N-methyltransferase, H3 lysine-9 specific 3	EHMT2	Writer	0.106166
Matrix metalloproteinase 10	MMP10	Protease	0.106166
C-X-C chemokine receptor type 7	ACKR3	Family A G protein-coupled receptor	0.106166
Tryptase beta-1	TPSAB1	Protease	0.106166
Hypoxanthine-guanine phosphoribosyltransferase	HPRT1	Enzyme	0.106166
Indoleamine 2,3-dioxygenase	IDO1	Enzyme	0.106166
Protein arginine N-methyltransferase 5	PRMT5	Writer	0.106166
Nicotinamide N-methyltransferase	NNMT	Enzyme	0.106166
Protein arginine N-methyltransferase 3	PRMT3	Writer	0.106166
Max-like protein X	MLX	Unclassified protein	0.106166
Geranylgeranyl transferase type I	PGGT1B FNTA	Enzyme	0.106166
Elastase 1	CELA1	Protease	0.106166
Pepsinogen C	PGC	Protease	0.106166
Sphingosine kinase 1	SPHK1	Enzyme	0.106166
Protein farnesyltransferase	FNTA FNTB	Enzyme	0.106166
Vasopressin V1a receptor	AVPR1A	Family A G protein-coupled receptor	0.106166
Immunoglobulin epsilon Fc receptor	FCER2	Membrane receptor	0.106166

Table S8. Prediction of potential targets of MDY peptides

Target	Common name	Target Class	Probability*
Mu opioid receptor (by homology)	OPRM1	Family A G protein-coupled receptor	0.13952343
Neurokinin 3 receptor	TACR3	Family A G protein-coupled receptor	0.104440651
Delta opioid receptor	OPRD1	Family A G protein-coupled receptor	0.095663487

Table S9. Prediction of potential targets of QIWYKSL peptides

Target	Common name	Target Class	Probability*
Mu opioid receptor (by homology)	OPRM1	Family A G protein-coupled receptor	0.458445
Somatostatin receptor 2	SSTR2	Family A G protein-coupled receptor	0.362778
HLA class I histocompatibility antigen A-3	HLA-A	Surface antigen	0.265597
Somatostatin receptor 4	SSTR4	Family A G protein-coupled receptor	0.2561
Delta opioid receptor	OPRD1	Family A G protein-coupled receptor	0.234596
Neurokinin 1 receptor	TACR1	Family A G protein-coupled receptor	0.189054
Neurotensin receptor 1 (by homology)	NTSR1	Family A G protein-coupled receptor	0.189054
Somatostatin receptor 3	SSTR3	Family A G protein-coupled receptor	0.160206
Ghrelin receptor	GHSR	Family A G protein-coupled receptor	0.122017
Endothelin receptor ET-A	EDNRA	Family A G protein-coupled receptor	0.112222
Somatostatin receptor 5	SSTR5	Family A G protein-coupled receptor	0.102432
Angiotensin-converting enzyme	ACE	Protease	0.102432
Cholecystokinin B receptor	CCKBR	Family A G protein-coupled receptor	0.102432
Cholecystokinin A receptor	CCKAR	Family A G protein-coupled receptor	0.092752
Membrane-associated guanylate	MAGI3	Enzyme	0.092752

kinase-related 3			
Melanocortin receptor 4	MC4R	Family A G protein-coupled receptor	0.083327
Melanocortin receptor 3	MC3R	Family A G protein-coupled receptor	0.083327
Chromobox protein homolog 7	CBX7	Reader	0.083327
Somatostatin receptor 1	SSTR1	Family A G protein-coupled receptor	0.083327
Neuropilin-1 (by homology)	NRP1	Secreted protein	0.083327
Kappa Opioid receptor	OPRK1	Family A G protein-coupled receptor	0.083327
C5a anaphylatoxin chemotactic receptor	C5AR1	Family A G protein-coupled receptor	0.073885
Renin	REN	Protease	0.073885
Melanocortin receptor 5	MC5R	Family A G protein-coupled receptor	0.073885
C3a anaphylatoxin chemotactic receptor	C3AR1	Family A G protein-coupled receptor	0.073885
Neprilysin	MME	Protease	0.073885
Cyclophilin A	PPIA	Isomerase	0.073885
Urotensin II receptor	UTS2R	Family A G protein-coupled receptor	0.073885
Vasopressin V2 receptor (by homology)	AVPR2	Family A G protein-coupled receptor	0.064239
Oxytocin receptor (by homology)	OXTR	Family A G protein-coupled receptor	0.064239
Vasopressin V1a receptor (by homology)	AVPR1A	Family A G protein-coupled receptor	0.064239
Matrix metalloproteinase 1	MMP1	Protease	0.064239
Matrix metalloproteinase 7	MMP7	Protease	0.064239
Tyrosine-protein kinase SRC	SRC	Kinase	0.064239
Matrix metalloproteinase 2	MMP2	Protease	0.064239
Histone deacetylase 3	HDAC3	Eraser	0.064239
Histone deacetylase 6	HDAC6	Eraser	0.064239
Histone deacetylase 3/Nuclear receptor corepressor 2 (HDAC3/NCoR2)	NCOR2 HDAC3	Eraser	0.064239
Calpain 1	CAPN1	Protease	0.064239
Matrix metalloproteinase 8	MMP8	Protease	0.064239
Proteasome subunit beta type-9	PSMB9	Enzyme	0.064239
Proteasome subunit beta type-8	PSMB8	Protease	0.064239
Neurotensin receptor 2	NTSR2	Family A G protein-coupled receptor	0.064239

CMP-N-acetylneuraminate-beta-galactosamide-alpha-2,3-sialyltransferase 1	ST3GAL1	Transferase	0.064239
Endothelin receptor ET-B	EDNRB	Family A G protein-coupled receptor	0.064239
Matrix metalloproteinase 3	MMP3	Protease	0.064239
Ephrin type-A receptor 2	EPHA2	Kinase	0.064239
Ephrin type-B receptor 2	EPHB2	Kinase	0.064239
Ephrin type-A receptor 5	EPHA5	Kinase	0.064239
Ephrin type-A receptor 4	EPHA4	Kinase	0.064239
Ephrin type-A receptor 8	EPHA8	Kinase	0.064239
Ephrin type-A receptor 6	EPHA6	Kinase	0.064239
Ephrin type-A receptor 7	EPHA7	Kinase	0.064239
Ephrin type-B receptor 3	EPHB3	Kinase	0.064239
Ephrin type-A receptor 3	EPHA3	Kinase	0.064239
Ephrin type-B receptor 1	EPHB1	Kinase	0.064239
Ephrin type-A receptor 1	EPHA1	Kinase	0.064239
Ephrin receptor	EPHB6	Unclassified protein	0.064239
Insulin-degrading enzyme	IDE	Enzyme	0.064239
Plasminogen	PLG	Protease	0.064239
Neurokinin 2 receptor	TACR2	Family A G protein-coupled receptor	0.064239
Peroxisome proliferator-activated receptor gamma	PPARG	Nuclear receptor	0.064239
Nociceptin receptor	OPRL1	Family A G protein-coupled receptor	0.064239
Neurokinin 3 receptor	TACR3	Family A G protein-coupled receptor	0.064239
Thrombin	F2	Protease	0.064239
Melanocortin receptor 1	MC1R	Family A G protein-coupled receptor	0.064239
Histone deacetylase 1	HDAC1	Eraser	0.064239
Inhibitor of apoptosis protein 3	XIAP	Other cytosolic protein	0.064239
Matrix metalloproteinase 9	MMP9	Protease	0.064239
Integrin alpha-IIb/beta-3	ITGA2B ITGB3	Membrane receptor	0.064239
Motilin receptor	MLNR	Family A G protein-coupled receptor	0.064239
Menin	MEN1	Unclassified protein	0.064239
Matrix metalloproteinase 16	MMP16	Protease	0.064239
ADAMTS5	ADAMTS5	Protease	0.064239
Matrix metalloproteinase 17	MMP17	Protease	0.064239
Matrix metalloproteinase 15	MMP15	Protease	0.064239
ADAM17	ADAM17	Protease	0.064239
Matrix metalloproteinase 14	MMP14	Protease	0.064239

Matrix metalloproteinase 26	MMP26	Protease	0.064239
ADAM10	ADAM10	Protease	0.064239
ADAM12	ADAM12	Protease	0.064239
ADAM9	ADAM9	Protease	0.064239
Epoxide hydratase	EPHX2	Protease	0.064239
Integrin alpha-4/beta-1	ITGB1 ITGA4	Membrane receptor	0.064239
Cathepsin (B and K)	CTSB	Protease	0.064239
Histone deacetylase 2	HDAC2	Eraser	0.064239
Matrix metalloproteinase 13	MMP13	Protease	0.064239
Matrix metalloproteinase 12	MMP12	Protease	0.064239
Integrin alpha-4/beta-7	ITGB7 ITGA4	Membrane receptor	0.064239
Cyclooxygenase-2	PTGS2	Oxidoreductase	0.064239
Protein farnesyltransferase	FNTA FNTB	Enzyme	0.064239
Cystinyl aminopeptidase	LNPEP	Protease	0.064239
Aminopeptidase N	ANPEP	Protease	0.064239
Histone deacetylase 8	HDAC8	Eraser	0.064239
SUMO-conjugating enzyme UBC9	UBE2I	Enzyme	0.064239
Serotonin transporter (by homology)	SLC6A4	Electrochemical transporter	0.064239
Baculoviral IAP repeat-containing protein 2	BIRC2	Enzyme	0.064239
Galanin receptor 1 (by homology)	GALR1	Family A G protein-coupled receptor	0.064239
Galanin receptor 2 (by homology)	GALR2	Family A G protein-coupled receptor	0.064239
Glucagon receptor (by homology)	GCGR	Family B G protein-coupled receptor	0.064239

Table S10. Prediction of potential targets of SKWKL peptides

Target	Common name	Target Class	Probability*
Neurotensin receptor 1	NTSR1	Family A G protein-coupled receptor	0.468908
Somatostatin receptor 2	SSTR2	Family A G protein-coupled receptor	0.296795
Somatostatin receptor 4	SSTR4	Family A G protein-coupled receptor	0.296795
Somatostatin receptor 1	SSTR1	Family A G protein-coupled receptor	0.258292
Mu opioid receptor (by homology)	OPRM1	Family A G protein-coupled receptor	0.239411
Somatostatin receptor 5	SSTR5	Family A G protein-coupled receptor	0.172499
Endothelin receptor ET-B	EDNRB	Family A G protein-coupled receptor	0.162959
HLA class I histocompatibility antigen A-3	HLA-A	Surface antigen	0.153392
Neurokinin 1 receptor	TACR1	Family A G protein-coupled receptor	0.153392
Somatostatin receptor 3	SSTR3	Family A G protein-coupled receptor	0.115101
Angiotensin-converting enzyme	ACE	Protease	0.105591
Histone deacetylase 2	HDAC2	Eraser	0.096027
Cyclophilin A	PPIA	Isomerase	0.096027

Epoxide hydratase	EPHX2	Protease	0.096027
Ghrelin receptor	GHSR	Family A G protein-coupled receptor	0.096027
Chromobox protein homolog 7	CBX7	Reader	0.086458
Endothelin receptor ET-A	EDNRA	Family A G protein-coupled receptor	0.086458
Histone deacetylase 3/Nuclear receptor corepressor 2 (HDAC3/NCoR2)	NCOR2 HDAC3	Eraser	0.07689
Histone deacetylase 11	HDAC11	Eraser	0.07689
Histone deacetylase 10	HDAC10	Eraser	0.07689
Delta opioid receptor	OPRD1	Family A G protein-coupled receptor	0.07689
Glucagon receptor (by homology)	GCGR	Family B G protein-coupled receptor	0.07689
Cholecystokinin B receptor	CCKBR	Family A G protein-coupled receptor	0.07689
C5a anaphylatoxin chemotactic receptor	C5AR1	Family A G protein-coupled receptor	0.067292
Matrix metalloproteinase 9	MMP9	Protease	0.067292
Membrane-associated guanylate kinase-related 3	MAGI3	Enzyme	0.067292
C3a anaphylatoxin chemotactic receptor	C3AR1	Family A G protein-coupled receptor	0.067292
Proteasome subunit beta type-9	PSMB9	Enzyme	0.067292
Proteasome subunit beta type-8	PSMB8	Protease	0.067292
Neuropilin-1 (by homology)	NRP1	Secreted protein	0.067292
Melanocortin receptor 4	MC4R	Family A G protein-coupled receptor	0.067292
Calpain 1	CAPN1	Protease	0.067292
Ephrin type-A receptor 2	EPHA2	Kinase	0.067292
Ephrin type-B receptor 2	EPHB2	Kinase	0.067292
Ephrin type-A receptor 5	EPHA5	Kinase	0.067292
Ephrin type-A receptor 4	EPHA4	Kinase	0.067292
Ephrin type-A receptor 8	EPHA8	Kinase	0.067292
Ephrin type-A receptor 6	EPHA6	Kinase	0.067292
Ephrin type-A receptor 7	EPHA7	Kinase	0.067292
Ephrin type-B receptor 3	EPHB3	Kinase	0.067292
Ephrin type-A receptor 3	EPHA3	Kinase	0.067292
Ephrin type-B receptor 1	EPHB1	Kinase	0.067292
Ephrin receptor	EPHB4	Kinase	0.067292
Ephrin type-A receptor 1	EPHA1	Kinase	0.067292
Ephrin receptor	EPHB6	Unclassified protein	0.067292
Cholecystokinin A receptor	CCKAR	Family A G protein-coupled receptor	0.067292
Histone deacetylase 3	HDAC3	Eraser	0.067292
Histone deacetylase 6	HDAC6	Eraser	0.067292
Histone deacetylase 4	HDAC4	Eraser	0.067292
Vasopressin V2 receptor (by homology)	AVPR2	Family A G protein-coupled receptor	0.057784
Vasopressin V1a receptor (by homology)	AVPR1A	Family A G protein-coupled receptor	0.057784

Oxytocin receptor (by homology)	OXTR	Family A G protein-coupled receptor	0.057784
Neprilysin (by homology)	MME	Protease	0.057784
Renin	REN	Protease	0.057784
Matrix metalloproteinase 7	MMP7	Protease	0.057784
Kappa Opioid receptor	OPRK1	Family A G protein-coupled receptor	0.057784
Matrix metalloproteinase 2	MMP2	Protease	0.057784
Matrix metalloproteinase 8	MMP8	Protease	0.057784
Matrix metalloproteinase 1	MMP1	Protease	0.057784
Histone deacetylase 1	HDAC1	Eraser	0.057784
Melanocortin receptor 3	MC3R	Family A G protein-coupled receptor	0.057784
Neurotensin receptor 2	NTSR2	Family A G protein-coupled receptor	0.057784
Peroxisome proliferator-activated receptor gamma	PPARG	Nuclear receptor	0.057784
Nociceptin receptor	OPRL1	Family A G protein-coupled receptor	0.057784
Melanocortin receptor 1	MC1R	Family A G protein-coupled receptor	0.057784
Histone deacetylase 8	HDAC8	Eraser	0.057784
Menin	MEN1	Unclassified protein	0.057784
Matrix metalloproteinase 16	MMP16	Protease	0.057784
ADAMTS5	ADAMTS5	Protease	0.057784
ADAMTS4	ADAMTS4	Protease	0.057784
Matrix metalloproteinase 17	MMP17	Protease	0.057784
Matrix metalloproteinase 15	MMP15	Protease	0.057784
ADAM17	ADAM17	Protease	0.057784
Matrix metalloproteinase 14	MMP14	Protease	0.057784
Matrix metalloproteinase 26	MMP26	Protease	0.057784
ADAM10	ADAM10	Protease	0.057784
ADAM12	ADAM12	Protease	0.057784
ADAM9	ADAM9	Protease	0.057784
Neurokinin 3 receptor	TACR3	Family A G protein-coupled receptor	0.057784
Cyclooxygenase-2	PTGS2	Oxidoreductase	0.057784
CMP-N-acetylneuraminate-beta-galactosamide-alpha-2,3-sialyltransferase 1	ST3GAL1	Transferase	0.057784
Galanin receptor 1 (by homology)	GALR1	Family A G protein-coupled receptor	0.057784
Galanin receptor 2 (by homology)	GALR2	Family A G protein-coupled receptor	0.057784
Cathepsin (B and K)	CTSB	Protease	0.057784
Matrix metalloproteinase 3	MMP3	Protease	0.057784
Melanocortin receptor 5	MC5R	Family A G protein-coupled receptor	0.057784
Motilin receptor	MLNR	Family A G protein-coupled receptor	0.057784
Neurokinin 2 receptor	TACR2	Family A G protein-coupled receptor	0.057784
Baculoviral IAP repeat-containing protein 2	BIRC2	Enzyme	0.057784
Protein farnesyltransferase	FNTA FNTB	Enzyme	0.057784
T-cell protein-tyrosine phosphatase	PTPN2	Phosphatase	0.057784

Plasminogen	PLG	Protease	0.057784
HLA class II histocompatibility antigen DRB1-1	HLA-DRB1	Surface antigen	0.057784
Leukotriene A4 hydrolase	LTA4H	Protease	0.057784
Intercellular adhesion molecule (ICAM-1), Integrin alpha-L/beta-2	ITGAL ICAM1 ITGB2	Membrane receptor	0.057784
Integrin alpha-4/beta-1	ITGB1 ITGA4	Membrane receptor	0.057784
Integrin alpha-4/beta-7	ITGB7 ITGA4	Membrane receptor	0.057784
Thrombin	F2	Protease	0.057784
Neuromedin B receptor	NMBR	Family A G protein-coupled receptor	0.057784
Inhibitor of apoptosis protein 3	XIAP	Other cytosolic protein	0.057784

Table S11. Prediction of potential targets of WQIWYK peptides

Target	Common name	Target Class	Probability*
Neurotensin receptor 1 (by homology)	NTSR1	Family A G protein-coupled receptor	0.169889
Mu opioid receptor (by homology)	OPRM1	Family A G protein-coupled receptor	0.169889
HLA class I histocompatibility antigen A-3	HLA-A	Surface antigen	0.131513
Delta opioid receptor	OPRD1	Family A G protein-coupled receptor	0.131513
C5a anaphylatoxin chemotactic receptor	C5AR1	Family A G protein-coupled receptor	0.102432
Ghrelin receptor	GHSR	Family A G protein-coupled receptor	0.102432
Endothelin receptor ET-A	EDNRA	Family A G protein-coupled receptor	0.092752
Cholecystokinin B receptor	CCKBR	Family A G protein-coupled receptor	0.092752
Kappa Opioid receptor	OPRK1	Family A G protein-coupled receptor	0.083327
Neurokinin 1 receptor	TACR1	Family A G protein-coupled receptor	0.083327
Cholecystokinin A receptor	CCKAR	Family A G protein-coupled receptor	0.083327
Angiotensin-converting enzyme	ACE	Protease	0.083327
Somatostatin receptor 2	SSTR2	Family A G protein-coupled receptor	0.083327
C3a anaphylatoxin chemotactic receptor	C3AR1	Family A G protein-coupled receptor	0.073885
Membrane-associated guanylate kinase-related 3	MAGI3	Enzyme	0.073885
Histone deacetylase 3	HDAC3	Eraser	0.073885
Histone deacetylase 6	HDAC6	Eraser	0.073885
Histone deacetylase 2	HDAC2	Eraser	0.073885
Histone deacetylase 1	HDAC1	Eraser	0.073885
Oxytocin receptor	OXTR	Family A G protein-coupled receptor	0.073885
Renin	REN	Protease	0.073885
Vasopressin V2 receptor (by homology)	AVPR2	Family A G protein-coupled receptor	0.073885
Cyclophilin A	PPIA	Isomerase	0.073885
Somatostatin receptor 5	SSTR5	Family A G protein-coupled receptor	0.064239

Somatostatin receptor 4	SSTR4	Family A G protein-coupled receptor	0.064239
Somatostatin receptor 1	SSTR1	Family A G protein-coupled receptor	0.064239
Somatostatin receptor 3	SSTR3	Family A G protein-coupled receptor	0.064239
Vasopressin V1a receptor (by homology)	AVPR1A	Family A G protein-coupled receptor	0.064239
Chromobox protein homolog 7	CBX7	Reader	0.064239
Matrix metalloproteinase 1	MMP1	Protease	0.064239
Matrix metalloproteinase 2	MMP2	Protease	0.064239
CMP-N-acetylneuraminate-beta-galactosamide-alpha-2,3-sialyltransferase 1	ST3GAL1	Transferase	0.064239
Histone deacetylase 3/Nuclear receptor corepressor 2 (HDAC3/NCoR2)	NCOR2 HDAC3	Eraser	0.064239
Histone deacetylase 8	HDAC8	Eraser	0.064239
Histone deacetylase 11	HDAC11	Eraser	0.064239
Histone deacetylase 10	HDAC10	Eraser	0.064239
Epoxide hydratase	EPHX2	Protease	0.064239
Matrix metalloproteinase 3	MMP3	Protease	0.064239
Melanocortin receptor 1	MC1R	Family A G protein-coupled receptor	0.064239
Urotensin II receptor	UTS2R	Family A G protein-coupled receptor	0.064239
Endothelin receptor ET-B	EDNRB	Family A G protein-coupled receptor	0.064239
Melanocortin receptor 3	MC3R	Family A G protein-coupled receptor	0.064239
Melanocortin receptor 4	MC4R	Family A G protein-coupled receptor	0.064239
Melanocortin receptor 5	MC5R	Family A G protein-coupled receptor	0.064239
Neurokinin 2 receptor	TACR2	Family A G protein-coupled receptor	0.064239
Neprilysin (by homology)	MME	Protease	0.064239
Calpain 1	CAPN1	Protease	0.064239
Histone deacetylase 4	HDAC4	Eraser	0.064239
Nociceptin receptor	OPRL1	Family A G protein-coupled receptor	0.064239
Neurotensin receptor 2	NTSR2	Family A G protein-coupled receptor	0.064239
Matrix metalloproteinase 9	MMP9	Protease	0.064239
Tyrosine-protein kinase SRC	SRC	Kinase	0.064239
Ephrin type-A receptor 2	EPHA2	Kinase	0.064239
Ephrin type-B receptor 2	EPHB2	Kinase	0.064239
Ephrin type-A receptor 5	EPHA5	Kinase	0.064239
Ephrin type-A receptor 4	EPHA4	Kinase	0.064239
Ephrin type-A receptor 8	EPHA8	Kinase	0.064239
Ephrin type-A receptor 6	EPHA6	Kinase	0.064239
Ephrin type-A receptor 7	EPHA7	Kinase	0.064239
Ephrin type-B receptor 3	EPHB3	Kinase	0.064239
Ephrin type-A receptor 3	EPHA3	Kinase	0.064239
Ephrin type-B receptor 1	EPHB1	Kinase	0.064239
Ephrin type-A receptor 1	EPHA1	Kinase	0.064239
Ephrin receptor	EPHB6	Unclassified protein	0.064239

Neurokinin 3 receptor	TACR3	Family A G protein-coupled receptor	0.064239
Matrix metalloproteinase 16	MMP16	Protease	0.064239
ADAMTS5	ADAMTS5	Protease	0.064239
Matrix metalloproteinase 17	MMP17	Protease	0.064239
Matrix metalloproteinase 15	MMP15	Protease	0.064239
ADAM17	ADAM17	Protease	0.064239
Matrix metalloproteinase 14	MMP14	Protease	0.064239
Matrix metalloproteinase 26	MMP26	Protease	0.064239
ADAM10	ADAM10	Protease	0.064239
ADAM12	ADAM12	Protease	0.064239
ADAM9	ADAM9	Protease	0.064239
Proteasome subunit beta type-9	PSMB9	Enzyme	0.064239
Proteasome subunit beta type-8	PSMB8	Protease	0.064239
Thrombin	F2	Protease	0.064239
Menin	MEN1	Unclassified protein	0.064239
Metastin receptor	KISS1R	Family A G protein-coupled receptor	0.064239
HLA class II histocompatibility antigen DRB1-1	HLA-DRB1	Surface antigen	0.064239
Motilin receptor	MLNR	Family A G protein-coupled receptor	0.064239
Peroxisome proliferator-activated receptor gamma	PPARG	Nuclear receptor	0.064239
Matrix metalloproteinase 8	MMP8	Protease	0.064239
Protein farnesyltransferase	FNTA FNTB	Enzyme	0.064239
Dipeptidyl peptidase IV	DPP4	Protease	0.064239
Plasminogen	PLG	Protease	0.064239
Integrin alpha-4/beta-1	ITGB1 ITGA4	Membrane receptor	0.064239
Integrin alpha-4/beta-7	ITGB7 ITGA4	Membrane receptor	0.064239
Cyclooxygenase-2	PTGS2	Oxidoreductase	0.064239
Serotonin transporter (by homology)	SLC6A4	Electrochemical transporter	0.064239
Inhibitor of apoptosis protein 3	XIAP	Other cytosolic protein	0.064239
Baculoviral IAP repeat-containing protein 2	BIRC2	Enzyme	0.064239
Neuropilin-1 (by homology)	NRP1	Secreted protein	0.064239
Cathepsin L	CTSL	Protease	0.064239
Matrix metalloproteinase 7	MMP7	Protease	0.064239
Leukotriene A4 hydrolase	LTA4H	Protease	0.064239
Ephrin receptor	EPHB4	Kinase	0.064239
Galanin receptor 1 (by homology)	GALR1	Family A G protein-coupled receptor	0.064239
Galanin receptor 2 (by homology)	GALR2	Family A G protein-coupled receptor	0.064239

Table S12. Prediction of potential targets of AFQLLNPK peptides

Target	Common name	Target Class	Probability*

HLA class I histocompatibility antigen A-3	HLA-A	Surface antigen	0.227733
Neurotensin receptor 1	NTSR1	Family A G protein-coupled receptor	0.102432
Neurotensin receptor 2	NTSR2	Family A G protein-coupled receptor	0.092752
Beta-secretase 1	BACE1	Protease	0.092752
CDK2/Cyclin A	CCNA2 CDK2	Kinase	0.092752
Mu opioid receptor	OPRM1	Family A G protein-coupled receptor	0.083327
Delta opioid receptor	OPRD1	Family A G protein-coupled receptor	0.083327
Thrombin	F2	Protease	0.083327
Renin	REN	Protease	0.083327
Angiotensin-converting enzyme	ACE	Protease	0.073885
Inhibitor of apoptosis protein 3	XIAP	Other cytosolic protein	0.073885
Cyclin-dependent kinase 4/cyclin D1	CCND1 CDK4	Kinase	0.064239
Cyclin-dependent kinase 2/cyclin A	CDK2 CCNA1 CCNA2	Other cytosolic protein	0.064239
Kappa Opioid receptor	OPRK1	Family A G protein-coupled receptor	0.064239
Vasopressin V2 receptor	AVPR2	Family A G protein-coupled receptor	0.064239
Beta secretase 2	BACE2	Protease	0.064239
Cathepsin D	CTSD	Protease	0.064239
Oxytocin receptor (by homology)	OXTR	Family A G protein-coupled receptor	0.064239
Thyrotropin-releasing hormone receptor (by homology)	TRHR	Family A G protein-coupled receptor	0.064239
Neurokinin 2 receptor	TACR2	Family A G protein-coupled receptor	0.064239
HLA class II histocompatibility antigen DRB3-1	HLA-DRB3	Surface antigen	0.064239
E3 SUMO-protein ligase CBX4	CBX4	Enzyme	0.064239
Endothelin receptor ET-A	EDNRA	Family A G protein-coupled receptor	0.064239
Histone deacetylase 3	HDAC3	Eraser	0.064239
Histone deacetylase 6	HDAC6	Eraser	0.064239
Neurotensin receptor 3	SORT1	Membrane receptor	0.064239
Aminopeptidase N	ANPEP	Protease	0.064239
Growth factor receptor-bound protein 2	GRB2	Other cytosolic protein	0.064239
MAP kinase ERK2	MAPK1	Kinase	0.064239
Melanocortin receptor 4	MC4R	Family A G protein-coupled receptor	0.064239

Melanocortin receptor 3	MC3R	Family A G protein-coupled receptor	0.064239
Kallikrein 1	KLK1	Protease	0.064239
Cyclooxygenase-2	PTGS2	Oxidoreductase	0.064239
C5a anaphylatoxin chemotactic receptor	C5AR1	Family A G protein-coupled receptor	0.064239
Ghrelin receptor	GHSR	Family A G protein-coupled receptor	0.064239
Cyclophilin A	PPIA	Isomerase	0.064239
Melanocortin receptor 5	MC5R	Family A G protein-coupled receptor	0.064239
Acyl-CoA: dihydroxyacetonephosphateacyltransferase	GNPAT	Enzyme	0.064239
Neprilysin	MME	Protease	0.064239
Neurokinin 1 receptor (by homology)	TACR1	Family A G protein-coupled receptor	0.064239
Cholecystokinin B receptor	CCKBR	Family A G protein-coupled receptor	0.064239
Tryptase beta-1	TPSAB1	Protease	0.064239
Chymotrypsin C	CTRC	Protease	0.064239
Neuropeptide FF receptor 1	NPFFR1	Family A G protein-coupled receptor	0.064239
Bradykinin B1 receptor	BDKRB1	Family A G protein-coupled receptor	0.064239
NAD-dependent deacetylase sirtuin 1	SIRT1	Eraser	0.064239
C3a anaphylatoxin chemotactic receptor	C3AR1	Family A G protein-coupled receptor	0.064239
Angiotensin II receptor	AGTR2	Family A G protein-coupled receptor	0.064239
Acidic mammalian chitinase	CHIA	Enzyme	0.064239
Calcitonin gene-related peptide type 1 receptor	CALCRL	Family B G protein-coupled receptor	0.064239
Tyrosine-protein kinase SRC	SRC	Kinase	0.064239
Disks large homolog 4	DLG4	Unclassified protein	0.064239
NAD-dependent deacetylase sirtuin 2	SIRT2	Eraser	0.064239
Neuromedin-U receptor 2	NMUR2	Family A G protein-coupled receptor	0.064239
Neuromedin-U receptor 1	NMUR1	Family A G protein-coupled receptor	0.064239
NAD-dependent deacetylase sirtuin 3	SIRT3	Eraser	0.064239
Vasopressin V1a receptor (by homology)	AVPR1A	Family A G protein-coupled receptor	0.064239
HMG-CoA reductase	HMGCR	Oxidoreductase	0.064239

Histone deacetylase 8	HDAC8	Eraser	0.064239
Lysine-specific histone demethylase 1	KDM1A	Eraser	0.064239
ADAM10	ADAM10	Protease	0.064239
Tubulin beta-1 chain	TUBB1	Structural protein	0.064239
Formyl peptide receptor 1	FPR1	Family A G protein-coupled receptor	0.064239
Chromobox protein homolog 7	CBX7	Reader	0.064239
Baculoviral IAP repeat-containing protein 3	BIRC3	Enzyme	0.064239
Integrin alpha-IIb/beta-3	ITGA2B ITGB3	Membrane receptor	0.064239
Neuropeptide FF receptor 2	NPFFR2	Family A G protein-coupled receptor	0.064239
Histone deacetylase 1	HDAC1	Eraser	0.064239
Cathepsin E	CTSE	Protease	0.064239
Cystinyl aminopeptidase	LNPEP	Protease	0.064239
Pepsinogen C	PGC	Protease	0.064239
Protein farnesyltransferase	FNTA FNTB	Enzyme	0.064239
Trypsin I	PRSS1	Protease	0.064239
Neuropeptide Y receptor type 4	NPY4R	Family A G protein-coupled receptor	0.064239
Proteinase-activated receptor 1	F2R	Family A G protein-coupled receptor	0.064239
Histone deacetylase 2	HDAC2	Eraser	0.064239
Histone deacetylase 11	HDAC11	Eraser	0.064239
Histone deacetylase 10	HDAC10	Eraser	0.064239
Serine/threonine-protein kinase AKT	AKT1	Kinase	0.064239
Somatostatin receptor 2	SSTR2	Family A G protein-coupled receptor	0.064239
Caspase-1	CASP1	Protease	0.064239
Somatostatin receptor 4	SSTR4	Family A G protein-coupled receptor	0.064239
Integrin alpha-V/beta-3	ITGAV ITGB3	Membrane receptor	0.064239
Histone deacetylase 3/Nuclear receptor corepressor 2 (HDAC3/NCoR2)	NCOR2 HDAC3	Eraser	0.064239
Integrin alpha-4	ITGA4	Membrane receptor	0.064239
Leucine aminopeptidase	LAP3	Protease	0.064239
Xaa-Pro dipeptidase	PEPD	Protease	0.064239
Xaa-Pro aminopeptidase 2	XPNPEP2	Protease	0.064239
Ribonucleoside-diphosphate reductase M1 chain	RRM1	Oxidoreductase	0.064239
Cyclin A2	CCNA2	Other cytosolic protein	0.064239
Proteasome Macropain subunit MB1	PSMB5	Protease	0.064239
C-X-C chemokine receptor type 7	ACKR3	Family A G protein-coupled receptor	0.064239

Matrix metalloproteinase 17	MMP17	Protease	0.064239
Caspase-2	CASP2	Protease	0.064239
Histamine H3 receptor	HRH3	Family A G protein-coupled receptor	0.064239
Pyroglutamylated RFamide peptide receptor	QRFPR	Family A G protein-coupled receptor	0.064239
Somatostatin receptor 3	SSTR3	Family A G protein-coupled receptor	0.064239
Matrix metalloproteinase 2	MMP2	Protease	0.064239
Matrix metalloproteinase 8	MMP8	Protease	0.064239
Protein-glutamine gamma-glutamyltransferase	TGM2	Enzyme	0.064239
